# Varifocal Alvarez metalens array for adaptive light-field imaging

**DOI:** 10.1038/s41467-026-74606-8

**Published:** 2026-06-23

**Authors:** Xiaoyu Che, Xiaoyuan Liu, Xin Zhang, Yuhao Lei, Yefeng Yu, Lei Wang, Junxiao Zhou, Zhishan Gao, Din Ping Tsai

**Affiliations:** 1 Department of Electrical Engineering and State Key Laboratory of Terahertz and Millimeter Waves, City University of Hong Kong, Kowloon, Hong Kong SAR China; 2 School of Electronic and Optical Engineering, Nanjing University of Science and Technology, Nanjing, China; 3 State Key Laboratory of Integrated Optoelectronics, College of Electronic Science and Engineering, Jilin University, Changchun, China; 4 Department of Physics and State Key Laboratory of Optical Quantum Materials, City University of Hong Kong, Kowloon, Hong Kong SAR China; 5 Shenzhen Research Institute, City University of Hong Kong, Shenzhen, China

**Keywords:** Nanophotonics and plasmonics, Photonic devices, Metamaterials, Imaging and sensing

## Abstract

Metalens arrays hold great promise for compact light-field imaging systems owing to their compactness and wavefront shaping capabilities. However, their fixed focal lengths fundamentally limit depth-of-field modulation and axial scanning, restricting their adaptability to diverse light-field imaging scenarios. Here, we demonstrate adaptive light-field imaging using a varifocal Alvarez metalens array. The array is composed of two closely bonded metasurfaces, each patterned with 24 × 20 cubic-phase sub-regions. Through relative lateral displacement, the Alvarez metalens array achieves continuous focal length tuning from 3.33 mm to 4.50 mm. Integrated into a plenoptic imaging system, the proposed metalens array enables dynamic focusing and an expanded depth range. We further introduce a MultiLensFusion algorithm that combines these multi-focus captures into high-resolution, all-in-focus renderings. This work offers a compact and versatile platform for next-generation light-field imaging systems with dynamically tunable optical response.

## Abstract (web summary)

Conventional lens arrays usually have fixed focal lengths. Here, the authors realize a 24 × 20 varifocal metalens array through lateral displacement of two metasurfaces, enabling adaptive light-field imaging without increasing system length.

## Introduction

Light-field imaging captures both spatial and angular information of light rays, enabling three-dimensional (3D) scene reconstruction. Plenoptic cameras, which incorporate a microlens array between a main lens and an image sensor, offer a compact architecture for encoding the 3D information^1,2^. However, the bulky form factor and fixed focal lengths of microlens arrays limit system integration and fundamentally restrict optical adaptability—the ability to dynamically modulate depth-of-field (DOF) and axial scanning to suit diverse imaging scenarios, which is essential for plenoptic and volumetric imaging^3,4^. Metalens arrays, composed of subwavelength-scale structures that manipulate spatially varying phase profiles for focusing light^5–7^, provide transformative advantages over traditional microlens arrays. They not only enable substantially reduced thickness and weight, but also unlock advanced optical capabilities such as tunability and on-chip integration^8–14^. These features make metalens arrays promising candidates for next-generation light-field imaging systems^15–21^. Yet, most reported metalens arrays still operate at a single fixed focal length, suffering from the lack of adaptability. Therefore, a varifocal metalens array is the key solution to achieve adaptive light-field imaging.

Existing varifocal metalenses employ tunable mechanisms such as flexible substrates^22,23^, phase change materials^24–28^, thermoelectric materials^29–31^, optical Kerr effect^32,33^, and external motion systems^34–45^. Among them, external-motion-systems-based varifocal designs are particularly notable for their wide tuning range and high precision in focal length adjustment, which are achieved through continuous relative motion between two constituent metasurfaces. The overall systems can be further miniaturized by integrating metasurfaces with micro-electromechanical systems (MEMS)^34,39^. Moiré metalenses and Alvarez metalenses represent two potential architectures for MEMS-based varifocal metalenses, driven by relative rotational motion and relative lateral displacement motion, respectively. Both of them can simultaneously achieve an ultra-thin form factor and a shortened optical path. While Moiré configurations are impractical for array-level rotation, Alvarez designs operate via a shared lateral displacement, making them highly compatible with array integration. An Alvarez-inspired varifocal metalens array thus presents a promising direction for innovation and may expand the functional scope of light-field imaging systems.

Optical adaptability is a critical design goal for light-field imaging systems. This capability includes not only extending the fixed DOF, which refers to the range of object space where the reconstructed scene appears acceptably sharp^46,47^, but also the complementary ability to dynamically adjust the DOF. Conventional strategies for extending DOF include interlaced arrays with different focal lengths^48^, sensor-shifting focal sweeping^49^, and bifocal metalens arrays combined with computational correction^50^. These methods either sacrifice pixel utilization or increase light path length and complexity. Moreover, in certain photographic contexts, such as portrait photography, a limited DOF is desirable to highlight the subject. In such cases, an extended DOF is not necessary; rather, an adaptive system with a tunable but limited DOF is preferred. Therefore, there is a compelling need for the development of a compact, lightweight varifocal light-field imaging device that can be adapted to diverse imaging scenarios.

In this work, we propose an Alvarez metalens array (AMA) that enables continuous focal length tuning from 3.33 mm to 4.50 mm through precise relative lateral displacement of two closely bonded metasurface layers from 0 to 69 μm, each patterned into 24 × 20 cubic-phase sub-regions. To validate the concept, the AMA is integrated into a plenoptic imaging system to realize dynamic optical adaptability without increasing the system length. We assess the imaging resolution at three different focal lengths, and then perform depth-resolved imaging experiments with two-dimensional (2D) objects placed at three corresponding focal planes. To produce an all-in-focus image, we develop a MultiLensFusion algorithm that fuses images captured at three different focal lengths. Finally, we validate the adaptive light-field imaging (ALI) performance on a complex 3D scene. The resulting all-in-focus 3D scene maintains high spatial resolution over an extended DOF. This demonstration highlights the potential of the compact plenoptic camera for versatile imaging applications and the AMA’s promise as a foundation for compact and ALI platforms.

## Results

### Alvarez metalens array design

**Fig. 1:**
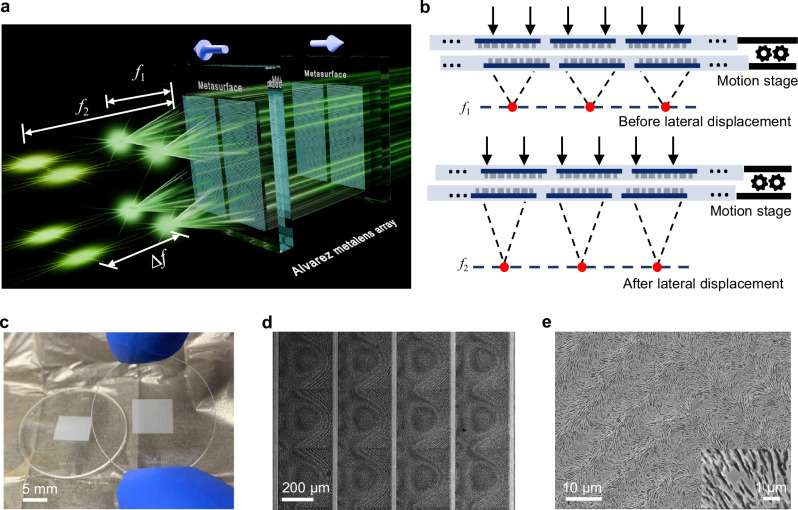
Schematic diagram and sample information of the AMA. **a** Schematic of a 2 × 2 AMA with tunable focal lengths from *f*_1_ to *f*_2_. The focal shift Δ*f* is induced by the relative lateral displacement between the metasurfaces (indicated by the blue arrows). **b** Side view of the focusing mechanism before and after lateral displacement, respectively. **c** Photography of the fabricated metasurfaces. **d** Optical microscope image showing a 3 × 4 array section of the fabricated metasurface. **e** SEM images of the anisotropic nanostructures at various magnifications.

The schematic of the proposed AMA is shown in Fig. 1a. Focal length tunability in a typical Alvarez metalens depends on the precise alignment of two metasurfaces (alignment error <1 μm), each encoded with 24 × 20 cubic phase profiles. The cubic terms are designed to have equal magnitudes but opposite signs, so that when the two metasurfaces are combined, the cubic components cancel, leaving a residual quadratic phase term that varies continuously with the relative lateral displacement. Because the phase profile of a thin lens is inherently quadratic, this variation in the quadratic term leads to a continuously tunable focal length. As illustrated in Fig. 1b, the focal length changes from *f*_1_ to *f*_2_ as the lateral displacement of each metasurface increases from 0 to *d*_max_, where *d*_max_ is half of the total maximum relative displacement. Under these conditions, the target phase profiles for the two metasurfaces can be expressed as^51^

1 $${\varphi }_{1}(x,y)=\frac{k}{12{d}_{\max }}\left(\frac{1}{{f}_{1}}-\frac{1}{{f}_{2}}\right){x}^{3}+\frac{k}{4{d}_{\max }}\left(\frac{1}{{f}_{1}}-\frac{1}{{f}_{2}}\right)x{y}^{2}-\frac{k}{4{f}_{1}}\left({x}^{2}+{y}^{2}\right),$$

2 $${\varphi }_{2}(x,y)=\frac{k}{12{d}_{\max }}\left(\frac{1}{{f}_{2}}-\frac{1}{{f}_{1}}\right){x}^{3}+\frac{k}{4{d}_{\max }}\left(\frac{1}{{f}_{2}}-\frac{1}{{f}_{1}}\right)x{y}^{2}-\frac{k}{4{f}_{1}}\left({x}^{2}+{y}^{2}\right),$$

where *k* denotes the wave vector in vacuum. Given the desired focal length range and maximum lateral displacement, the phase profiles can be directly computed from Eqs. (1, 2). The relationship between the focal length and the displacement *d* of each metasurface can be described by

3 $$\frac{1}{f}=\frac{1}{{f}_{1}}-\left(\frac{1}{{f}_{1}}-\frac{1}{{f}_{2}}\right)\frac{d}{{d}_{\max }},$$

where *d* denotes the lateral displacement of each metasurface. The normalized displacement, or displacement ratio, is defined as *d*/*d*_max_, varying from 0 to 1 throughout the tuning process. For a given focal length range, the focal length depends solely on this displacement ratio.

For a proof-of-concept demonstration, we design and fabricate a 24 × 20 AMA. As shown in Fig. 1c, each sub-metalens occupies an area of 345 μm × 345 μm, yielding a total metasurface pattern size of 8.28 mm × 6.9 mm. The metasurfaces are embedded within one-inch-diameter silica wafers with a thickness of 1 mm. Fabrication is performed using a femtosecond-pulse laser to inscribe the metasurface patterns approximately 100 μm beneath the wafer surface^52^, providing subsurface protection during both assembly and lateral displacement. Detailed fabrication procedures are described in the “Methods” section. Within each sub-region, an unpatterned stripe with a width of 34.5 μm is reserved, as shown in Fig. 1d, to accommodate the lateral displacement between the two metasurfaces. A scanning electron microscopy (SEM) image is shown in Fig. 1e. The nanostructures within the metasurface pattern area are anisotropic, exhibiting polarization-dependent responses at the wavelength scale. Specifically, their effective phase response differs under *x*-polarized light and *y*-polarized light, inducing a Pancharatnam-Berry phase manipulation for the circularly polarized light^53^.

**Fig. 2:**
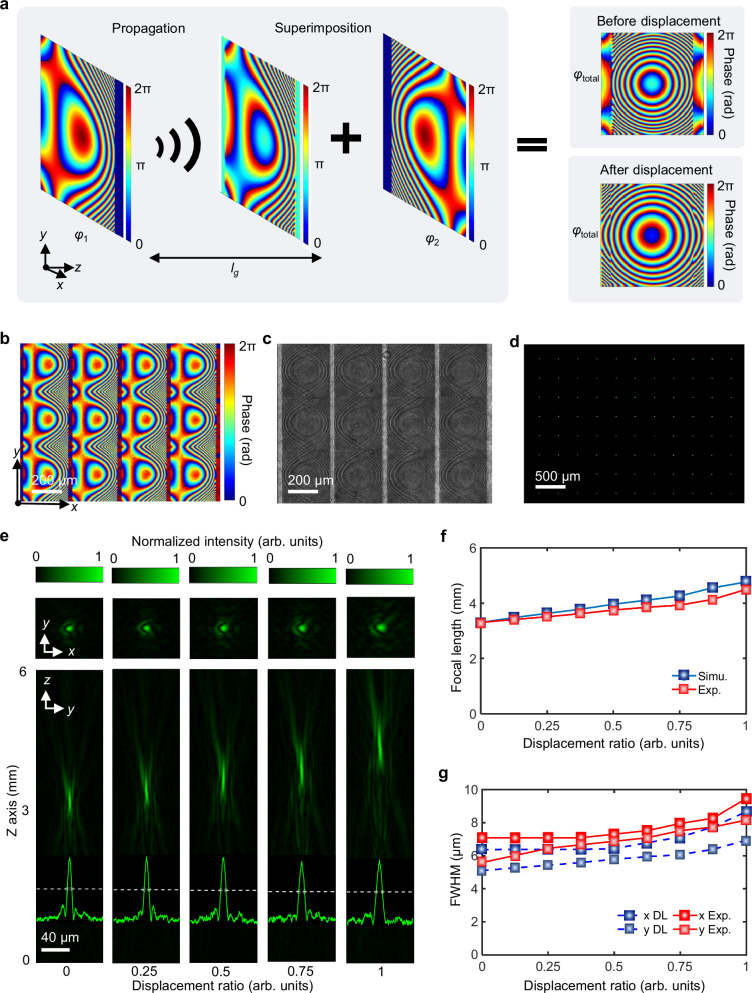
Simulated and measured optical performance of the fabricated metalens array. **a** Simulated total phase profile of a single Alvarez metalens before and after lateral displacement. **b** Experimentally measured phase profile of a portion of the metasurface. **c** Optical microscope image of a 3 × 4 section of the AMA at the displacement ratio of 0.5. **d** A portion of the focal spots array generated by the AMA under normally incident circularly polarized light at a wavelength of 532 nm. **e** Measured intensity distribution of a single focal spot in the xy-plane and yz-plane at different displacement ratios. The intensity profiles along the center line of the focal plane are marked by the green lines, and the positions of the FWHMs are marked by the white dashed lines. **f** Simulated and experimental focal lengths of the AMA at various displacement ratios. **g** FWHMs of the AMA at different displacement ratios. The ‘DL’ represents the diffraction limit, the red line represents experimental (Exp.) results, and the blue line represents simulated (Simu.) results.

The theoretically calculated focal length of the designed AMA ranges from 3.3 mm to 4.8 mm, corresponding to a relative displacement range from 0 to 69 μm. The phase profiles of the two metasurfaces, denoted as *φ*_1_ and *φ*_2_, under illumination at a wavelength of 532 nm, are obtained from Eqs (1, 2), and illustrated in Fig. 2a. Incident light interacts with the initial metasurface, propagates a distance *l*_g_ between the two metasurfaces, and then impinges on the second metasurface. During this free-space propagation, the phase profile is altered from the ideal phase *φ*_1_ before reaching the second metasurface. The resulting total phase distribution, *φ*_total_, is computed using the Rayleigh-Sommerfeld diffraction method.

4 $${\varphi }_{{{{\rm{total}}}}}={{\mbox{arg}}}\{[\exp (i{\varphi }_{1}) * h(x,y,{l}_{g})]\cdot \exp (i{\varphi }_{2})\},$$

where *h*(*x*,*y*,*l*_*g*_) is the free-space propagation function over the distance *l*_g_ between the two metasurfaces. In this work, *l*_g_ is approximately 200 μm, which is a balanced decision based on fabrication feasibility and optical performance, as detailed in Supplementary Information I. The simulated total phase profiles exhibit slight deviations from the ideal ones, leading to focal length errors and distortions of the focal spots.

To demonstrate the phase modulation capability and structural fidelity of the fabricated metasurface, we perform phase imaging measurements using a high-resolution wavefront sensor camera (SID4, PHASICS). The measured phase profile of a 3 × 4 sub-section of the metalens array shown in Fig. 2b agrees well with the theoretical design in Fig. 2a. The optical performance of the generated focal spots is characterized using a point spread function (PSF) measurement setup, as illustrated in Supplementary Information I. The metasurfaces are mounted on independent translation stages and precisely aligned to assemble the AMA. A collimated circularly polarized laser beam (*λ* = 532 nm) is normally incident on the AMA. Figure 2c displays a 3 × 4 section of the assembled array with completely overlapped unpatterned stripes, corresponding to a displacement ratio of 0.5.

The PSF measurement system collects the light intensity distribution along the optical axis, where the focal plane is defined at the maximum intensity point. At this plane, an 8 × 11 array of focal spots is shown in Fig. 2d. The normalized intensity distribution of a magnified single focal spot in the xy- and yz-planes is presented in Fig. 2e. By continuously tuning the displacement ratio from 0 to 1, the measured focal length varies smoothly from 3.33 mm to 4.50 mm (see Supplementary Movie 1). Simulated and experimental focal length data are shown in Fig. 2f. The maximum deviation in focal length is less than 7%, attributed to fabrication imperfections and alignment errors.

Figure 2g presents the full-width at half-maximum (FWHM) of the focal spots, extracted from the normalized intensity profiles in the xz- and yz-planes. Both values are slightly larger than the theoretical diffraction-limited FWHM. The difference between the measured and simulated FWHMs is primarily due to focal length deviations, while the FWHM asymmetry between the two planes results from the anisotropic aperture geometry of the sub-metalenses. Specifically, lateral displacement modifies not only the focal length, but also the aperture width of the sub-metalenses (from 276 μm to 310.5 μm and back to 276 μm). These two factors collectively determine the numerical aperture (NA) along the xz-plane focusing, which gradually decreases from 0.052 to 0.038.

### Implementation of ALI with proposed AMA

**Fig. 3:**
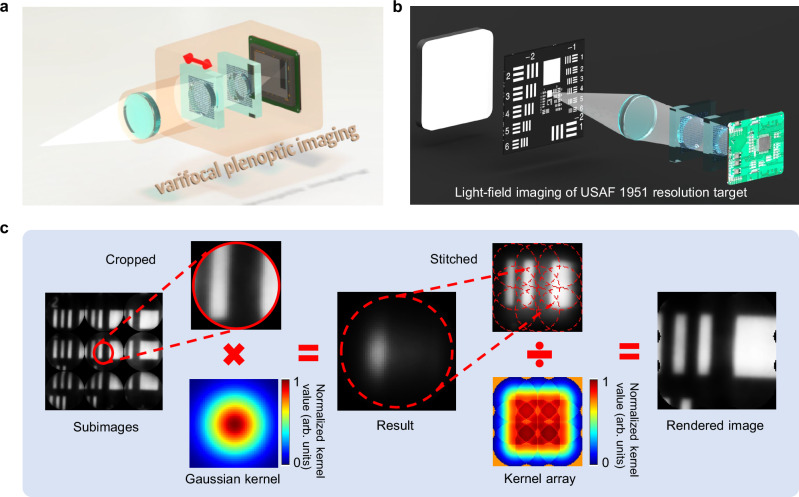
Optical design and image reconstruction workflow of the ALI. **a** Schematic of the proposed ALI system. **b** Diagram of the light-field imaging of a USAF 1951 resolution target. **c** Illustration of the disparity estimation and sub-image stitching.

Employing the AMA, we construct a proof-of-concept ALI system that can focus on objects outside the depth-of-field (DOF) of the traditional plenoptic camera through relative lateral displacement between the componential metasurfaces. With careful optical design (see details in Supplementary Information II), the ALI system achieves a continuously tunable focal plane ranging from 1 m to 5 m, while the DOF with a fixed focal length cannot cover the entire object field. The design procedure and the simulated optical performance are shown in the supplementary material. The schematic diagram of the ALI system is shown in Fig. 3a. The AMA is placed slightly away from the focal length of the achromatic main lens and the image sensor, re-imaging the main lens image and creating a sub-image array onto the image sensor. The main lens also provides the entrance pupil, which defines the circular aperture boundaries of the sub-images. The image sensor is dimensionally matched to the AMA, and its pixel size is comparable to the focus spot generated by the ALI system.

To evaluate the imaging resolution performance of the designed ALI system at different focal settings, a USAF 1951 resolution target is placed at distances of 1.25 m, 2.5 m, and 5 m from the main lens. The target is uniformly illuminated by a distant white-light panel to ensure consistent lighting conditions, as shown in Fig. 3b. When the ALI system is focused at each of the specified distances, the corresponding DOF ranges remain distinct and non-overlapping, thereby avoiding interference between different imaging planes.

Captured light-field sub-images are processed using image stitching and reconstruction algorithms to obtain high-resolution rendered images at each focal setting, enabling a comprehensive assessment of the system’s adaptive imaging capability. The preprocessing of light field white images involves extracting the metalens positions and grid structures by segmenting a calibration image captured under uniform illumination. The detected regions are then refined using morphological operations and thresholding. Next, a grid geometric regularization is applied to correct distortions, ensuring accurate alignment of metasurface positions for subsequent light field image analysis (see details in Fig. S9).

Disparity for each metalens is estimated by comparing it with its eight nearest neighbors, constructing a cost volume via a plane-sweep approach. The averaged cost volume is regularized using Semi-Global Matching to generate the final disparity and confidence maps for all sub-metalenses (see details in Fig. S10). As shown in Fig. 3c, the rendering process involves extracting patches from each metalens, with their sizes determined by the disparity value, balanced using both confidence-based and edge-based methods. These patches are stitched together to render the final image. Circular masks and Gaussian weighting are applied to suppress boundary artifacts and improve blending at patch boundaries. Final contributions from overlapping regions are normalized using the accumulated Gaussian weights. Compared to square masks or rendering without Gaussian weighting, this approach ensures smoother transitions and consistent brightness across the image.

**Fig. 4:**
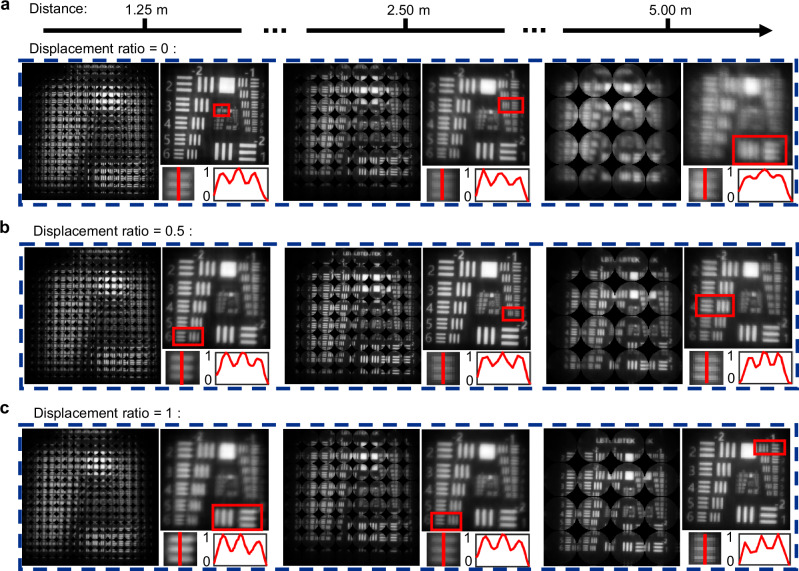
Resolution evaluation of the ALI. Grayscale sub-images of a USAF 1951 resolution target captured by the ALI system with focal lengths (**a**) 3.33 mm, **b** 3.75 mm, and **c** 4.50 mm, and the corresponding rendered image at depths of 1.25 m, 2.5 m, and 5 m. The corresponding zoom-in images in the red boxes and the intensity cross sections of the smallest resolvable line pairs along the red lines are shown in the lower right.

The light-field sub-images and the corresponding rendered images of the resolution target are shown in Fig. 4a–c, with the smallest resolvable line pairs indicated. The imaging results here are converted to grayscale images. Resolution is evaluated according to the Rayleigh criterion, which defines resolvability when the intensity ratio of the central minimum to the adjacent maximum in the cross-sectional intensity distribution of each line pair is less than 0.736.

At zero lateral displacement, the resolution target at 1.25 m lies within the DOF of the ALI, yielding the smallest resolvable spatial frequency of 1.122 lp/mm (Group 0, Element 2). For comparison, the rendered images of resolution targets placed at 2.5 m and 5 m are also shown in Fig. 4a, corresponding to the smallest resolvable spatial frequency of 0.63 lp/mm (Group −1, Element 3) and beyond the measurable limit, respectively. When the displacement ratio is adjusted to 0.5 and 1, the resolution targets at 2.5 m and 5 m come into focus, and the smallest resolvable line pairs are 0.891 lp/mm (Group −1, Element 6) and 0.5 lp/mm (Group −1, Element 1), respectively. The decrease in resolvable spatial frequency for farther objects is expected, as they inherently suffer greater loss in spatial resolution, as also seen in Fig. 4b, c. Although the chromatic aberration of the AMA introduces slight blurring even in focus, the resolution remains adequate for light-field rendering.

**Fig. 5:**
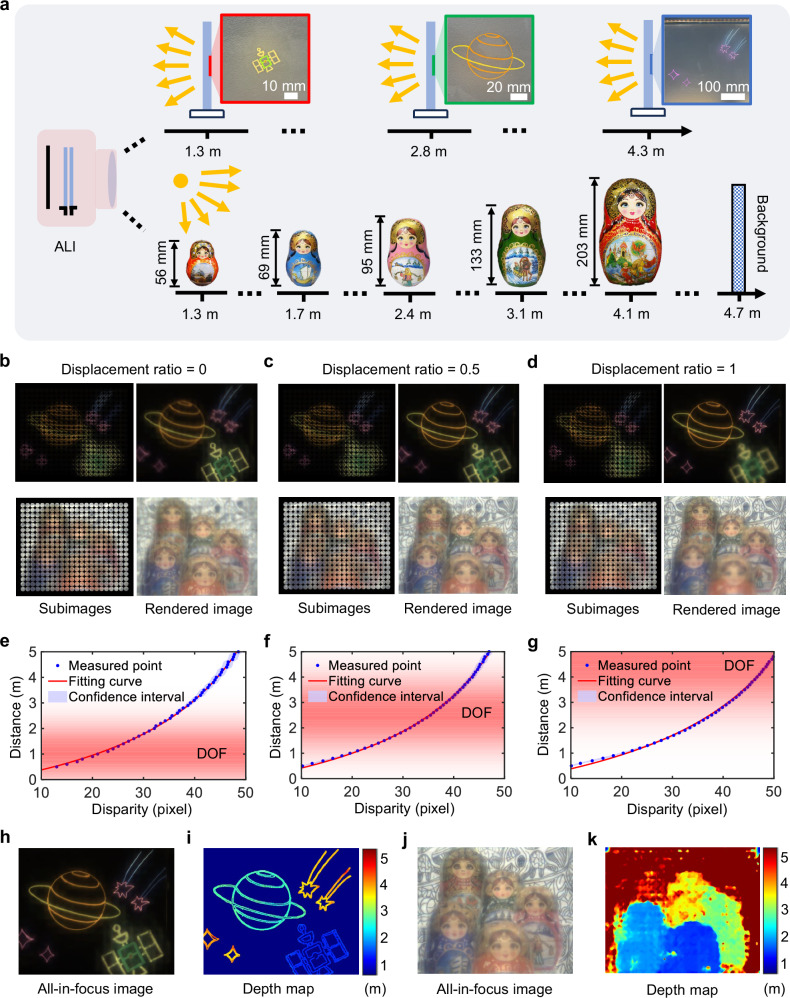
Experiments of the ALI. **a** Light-field image of the fluorescent lamp boards and nesting dolls at different distances. The satellite, the planet, and the stars are at distances 1.3 m, 3.0 m, and 4.3 m, respectively. The orange, blue, pink, green, and red nesting dolls are at distances 1.3 m, 1.7 m, 2.4 m, 3.1 m, and 4.1 m. **b–d** The light-field sub-images and the corresponding rendered images with different displacement ratios 0, 0.5, and 1. **e–g** The tested disparities at different distances with different displacement ratios 0, 0.5, and 1. The red background indicates the DOF range. **h**,** j** The all-in-focus images of the two scenes. **i**,** k** Depth estimation of the scenes.

To further evaluate the depth estimation capability of the proposed ALI, we conduct imaging experiments using both 2D and 3D objects positioned at different depths. For the 2D case, three fluorescent boards with integrated light sources are used, each featuring a hand-drawn pattern of a satellite, a planet, and several stars. The boards are placed at 1.3 m, 2.8 m, and 4.3 m from the main lens, respectively, as shown in Fig. 5a, and are designed for visibility under dark ambient conditions. For the 3D case, a set of colored nesting dolls is sequentially placed at 1.3 m, 1.7 m, 2.4 m, 3.1 m, and 4.1 m from the main lens. The entire scene is arranged in front of a background board fixed at 4.7 m and illuminated under bright illumination to ensure high contrast for imaging.

Light-field sub-images and their corresponding rendered images at displacement ratios of 0, 0.5, and 1 are shown in Fig. 5b–d. These rendered results clearly demonstrate that the effective DOF shifts from near to far with increasing lateral displacement. Object depths are estimated from the captured scenes using a scanning parallax calibration method, as detailed in Supplementary Information II. The theoretical relation between disparity and depth can be described as

5 $$\frac{1}{l}=\frac{1}{{f}_{L}}-\frac{1}{{B}_{L}-B+\frac{BD}{2\cdot {{{\rm{Disparity}}}}\cdot p}},$$

where *l* denotes the distance from main lens to object, *f*_*L*_ is the focal length of the main lens, *D* is the diameter of sub-metalens, *B*_*L*_ represents the distance from the main lens to the AMA, *B* is the distance between the varifocal metalens array to image sensor, and *p* denotes the pixel size of the image sensor (calculated parameters are shown in Supplementary Information II).

During lateral displacement, the measured disparities at various object depths ranging from 0.5 m to 5 m are shown in Fig. 5e–g. The experimental data points closely follow the theoretical disparity-depth curve within the corresponding DOF. Each data point is obtained from ten repeated measurements. Data points falling outside the DOF are excluded due to increased fitting errors. Based on the fitted curve, all-in-focus images of the scenes are reconstructed using a MultiLensFusion algorithm. This algorithm integrates precise metalens-center correction, confidence- and edge-guided disparity estimation, Gaussian-weighted blending, and sharpness-based rendering (see Supplementary Information III for details). The resulting all-in-focus images are presented in Fig. 5h, j, along with the corresponding depth maps shown in Fig. 5i, k.

## Discussion

Inspired by the Alvarez lens design, we demonstrate an AMA with a continuously tunable focal length ranging from 3.33 mm to 4.50 mm. This tunability is achieved through a relative lateral displacement between two metasurfaces. The metasurfaces are fabricated using a laser writing method in a silica substrate, which helps protect the nanostructures from potential damage during alignment. Alternatively, electron-beam lithography can be employed to design isotropic meta-atoms, enabling polarization-insensitive operation under natural light without additional polarization optics.

The full potential of this adaptive system can be unlocked by integrating the AMA with compact driving mechanisms, such as piezoelectric actuators or MEMS, for fast and precise focal tuning^39^. The AMA can also incorporate polarization-dependent dual-focus capabilities while maintaining continuous varifocal operation^44^. For ultimate miniaturization, one metasurface could be integrated directly onto the image sensor^13^. Such miniaturized devices hold strong potential for 3D reconstruction, parallel nanofabrication^45,54^, and adaptive optics.

Furthermore, we demonstrate ALI enabled by the proposed AMA, achieving an extended DOF through a MultiLensFusion algorithm. Critically, the ALI system can be coupled with an image feedback system to automatically focus selectively at target depths, realizing a truly complete adaptive optical system. While chromatic aberration remains a limitation, it can be mitigated via an achromatic metasurface design^55^ or a neural network reconstruction algorithm^50^. In conclusion, our ALI prototype provides a compact and reconfigurable architecture for light-field imaging. This demonstrated dynamic optical adaptability holds strong potential for applications requiring compact, dynamic 3D imaging, such as drone-based aerial imaging, medical endoscopy, surgical navigation, and intelligent surveillance.

## Methods

### Nanofabrication of the metalens array

To demonstrate the proposed concept, we designed two dielectric metasurfaces that were then fabricated by ultrafast laser writing. The laser writing experiments were carried out with a Yb:KGW femtosecond laser system (Carbide, Light Conversion Ltd.), operating at 1030 nm and delivering pulses with a 200 kHz repetition rate and a duration of 500 fs. The laser beam was focused via a 0.16 numerical aperture (NA) aspheric lens beneath the surface of a synthetic silica glass plate, which was mounted on a XYZ linear air-bearing translation stage (Aerotech Ltd.) for precise positioning. The single pulse energy was 1.5 μJ, and the focused beam radius was 2.7 μm, leading to a fluence of 6.5 J/cm^2^ and an intensity of 13 TW/cm^2^. During laser writing, the sample was translated at a speed of 3 mm/s, resulting in a pulse density of about 66 pulses/μm. The distance between the lines was fixed at 1 μm. A combination of a polarizer, an electro-optic modulator, and a quarter-wave plate was used to control the polarization azimuth of the laser beam. The retardance and slow-axis orientation of the birefringent modifications were quantified using a birefringence measurement system (Oosight imaging system, Hamilton Thorne) operating at 546 nm and built into an Olympus BX53 optical microscope.

Moreover, during high-intensity femtosecond laser writing, tightly focused ultrashort pulses generate a high-density free-electron plasma inside silica glass. The interaction between the incident light field and the plasma produces a periodic electric-field modulation, which gradually stabilizes under multi-pulse irradiation and leads to the formation of self-assembled nano-gratings^56^. Because the grating period is far below the incident wavelength, direct writing of subwavelength structures can be achieved. The period of such nano-gratings was approximately 350 nm, which was close to *λ*_*w*_/2*n*, where *λ*_*w*_ was the wavelength of the writing laser beam (1030 nm), and *n* was the refractive index of silica glass at 1030 nm.

### Characterization of the metalens array

The experimental setup for characterizing the focal spots of the metalens array is detailed in the Supplementary Information I. A collimated 532-nm laser beam, incident on a polarizer and quarter-wave plate, generates circularly polarized illumination. The beam impinges orthogonally on the metalens array plane. The focal spots generated by the metalens array are captured by a 20X objective (NA = 0.42) coupled to an image sensor (MV-CB050-11UC-C, HIKROBOT).

For nano-structural characterization, the laser-processed glass was lapped and polished to reveal the modified region, followed by chemical etching in a 1% hydrofluoric (HF) acid solution for 5 s. The etched surfaces were subsequently imaged using a scanning electron microscope (SEM, Zeiss EVO 50).

## Supplementary information

Supplementary Information

Description of Additional Supplementary Files

Supplementary Movie 1

Transparent Peer Review file

## Data Availability

The data that support the findings of this study are available from the corresponding author upon request.

## Code availability

The code used for analyses and figures is available from the corresponding author upon request.
