# Supplementary material for: Varifocal Alvarez metalens array for adaptive light-field imaging
Source: Nat Commun. 2026 Jun 23;17:7882. doi: 10.1038/s41467-026-74606-8 (PMC13442863; doi:10.1038/s41467-026-74606-8)
Supplement: Supplementary file 1 [file 41467_2026_74606_MOESM1_ESM.pdf]

# Supplementary information

## Varifocal Alvarez metalens array for adaptive light-field imaging


Xiaoyu Che[1,2,†], Xiaoyuan Liu[1,†], Xin Zhang[3], Yuhao Lei[3], Yefeng Yu[2], Lei Wang[3], Junxiao Zhou[1,*], Zhishan Gao[2,*], and Din Ping Tsai[1,4,5,*]

1. *Department of Electrical Engineering and State Key Laboratory of Terahertz and Millimeter Waves, City University of Hong Kong, Kowloon, Hong Kong SAR 999077, China*

2. *School of Electronic and Optical Engineering, Nanjing University of Science and Technology, Nanjing, 210094, China*

3. *State Key Laboratory of Integrated Optoelectronics, College of Electronic Science and Engineering, Jilin University, Changchun, 130012, China*

4. *Department of Physics and State Key Laboratory of Optical Quantum Materials, City University of Hong Kong, Kowloon, Hong Kong SAR 999077, China*

5. *Shenzhen Research Institute, City University of Hong Kong, Shenzhen, 518057, China*

* *junxiaozhou@nju.edu.cn*

* *zhishgao@njust.edu.cn*

* *dptsai@cityu.edu.hk*

†. *These authors contributed equally: Xiaoyu Che and Xiaoyuan Liu*

*. *corresponding authors: Junxiao Zhou, Zhishan Gao, Din Ping Tsai*

# I Design of the Alvarez metalens array

## 1. Principles of phase manipulation by nanostructures

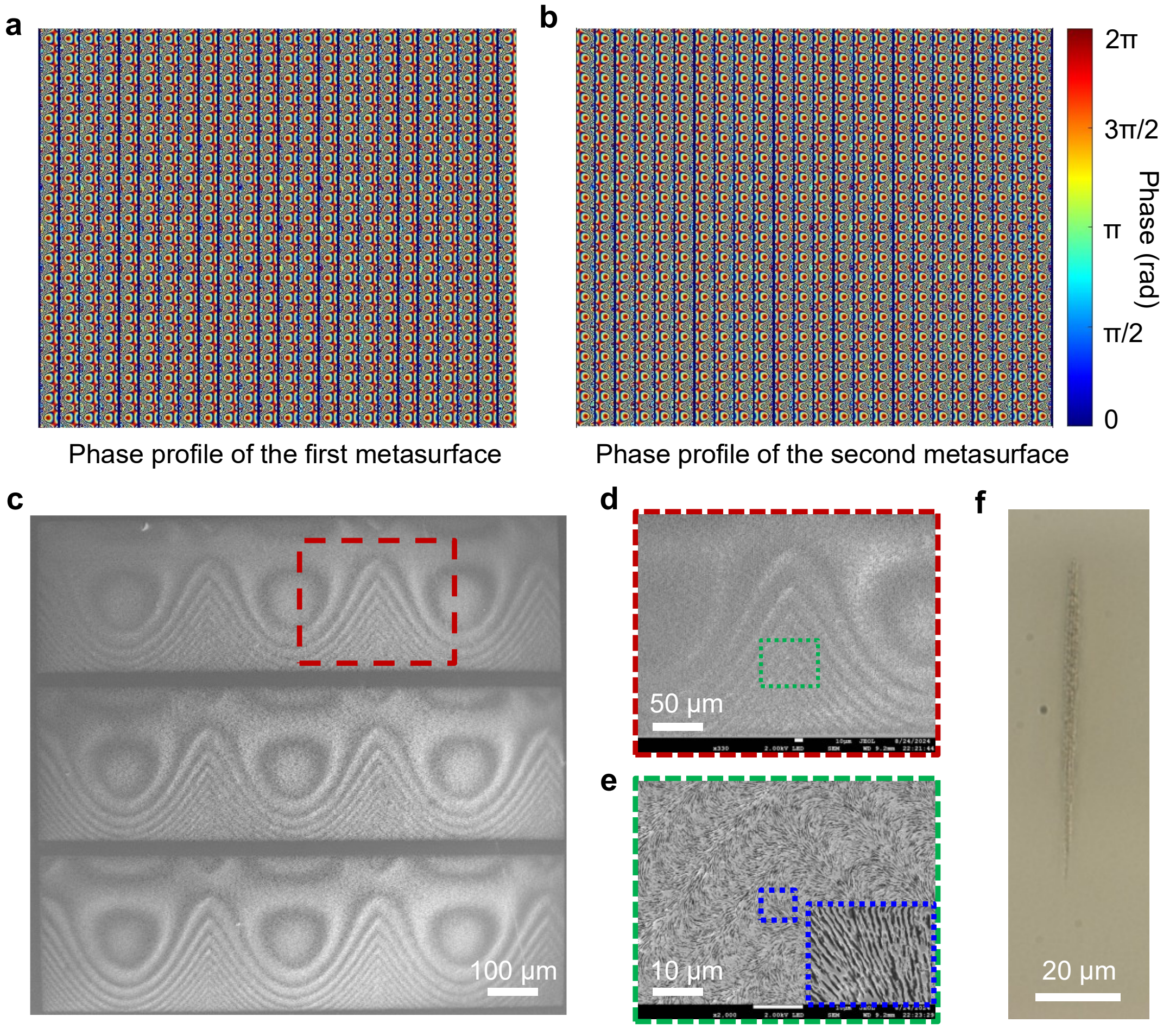


**Figure S1 Phase profiles and images of the fabricated metasurfaces.** The phase profiles of **a** the first metasurface and **b** the second metasurface. Scanning electron microscope (SEM) images of the fabricated metasurface with **c** 50 X, **d** 330 X, and **e** 2000 X magnification, respectively. **f** Morphology of the nanostructures in the depth direction observed by an optical microscope.

The designed metasurface contains 24×20 sub-regions, as shown in Fig. S1a and S1b. The relative displacement between the first and second metasurfaces induces a corresponding displacement between the associated sub-regions. Each pair of aligned sub-regions forms a sub-metalens. The nanostructures are fabricated using a laser writing technique, as shown in Fig. S1c-e. The fundamental reason that these structures can achieve sub-100 nm feature sizes lies in the fact that the fabrication mechanism does not rely on the optical focusing resolution of the writing laser, but is instead governed by the strong nonlinear ionization and plasma dynamics induced by femtosecond

laser irradiation in fused silica. The fabricated structures in this work consist of self-organized nano-gratings composed of periodically arranged nano-slots, whose geometrical scale can be precisely controlled by tuning the femtosecond laser writing parameters. The grating period is strongly dependent on the laser conditions: it decreases with increasing pulse number, while it increases with higher single-pulse energy. Under typical conditions, the period of such nano-gratings is approximately 350 nm. Notably, each grating period contains multiple nano-slot substructures (typically around ten), with an individual nano-slot width of approximately 20 nm, enabling sub-100 nm local feature dimensions.

In terms of functionality, the nanostructures operate based on an effective birefringent mechanism, with the design target of achieving a π phase retardation, corresponding to a half-wave plate response. Under this condition, precise propagation phase accumulation is achieved by controlling the anisotropic structural parameters of the nano-gratings, including nano-slot density, morphological uniformity, and effective birefringence, as well as the effective propagation thickness. When the phase retardation reaches exactly π, complete conversion of the incident linear polarization state can be realized, corresponding to an ideal polarization conversion efficiency approaching 100%. Therefore, the optimization of fabrication accuracy and operational efficiency is intrinsically unified in the precise control of the self-organized nanostructure uniformity and the resulting effective birefringent phase retardation, ensuring that the device performance closely approaches the ideal half-wave plate condition for efficient optical field manipulation.

These anisotropic nano-gratings manipulate the Pancharatnam-Berry (PB) phase of the incident light. The phase modulation is governed by the collective rotation angle $\theta$ of nano-gratings within a subwavelength scale region. The transmitted electric field can be described as

$$\mathbf{E} = t_1 \cdot \hat{\mathbf{e}} + t_2 \mathrm{e}^{-i2\theta} \cdot \hat{\mathbf{e}}^*,$$

where $\hat{\mathbf{e}} = [1\ \sigma i]^{\mathrm{T}}$, $\sigma = \pm 1$ denotes the polarization state of the electric field, $t_1$ and $t_2$ are the complex transmission coefficients and complex polarization conversion coefficients, respectively, $*$ represents complex conjugation, and $-2\theta$ represents the induced PB phase. When the polarization conversion efficiency $|t_2^* t_2|$ is sufficiently high, the first term becomes negligible. Under this condition, a circularly polarized beam passing through the first metasurface undergoes a polarization state transformation into its orthogonal counterpart, accompanied by PB phase modulation. Upon

transmission through the second metasurface, the light returns to its polarization state, acquiring an additional PB phase shift in the process.

Regarding the morphology in the depth direction, the nanostructures form an elongated ellipsoid. This morphology is dictated by the 3D focal volume of the objective lens used for the femtosecond laser direct writing. Because the axial Rayleigh length of the focused Gaussian beam is larger than its lateral waist, the induced plasma and subsequent nanograting self-assembly extend along the beam propagation axis. Figure S1f shows the morphology of the nanostructures in the depth direction, which are fabricated by a single focused laser beam.

## 2 Phase characterization of the metasurface

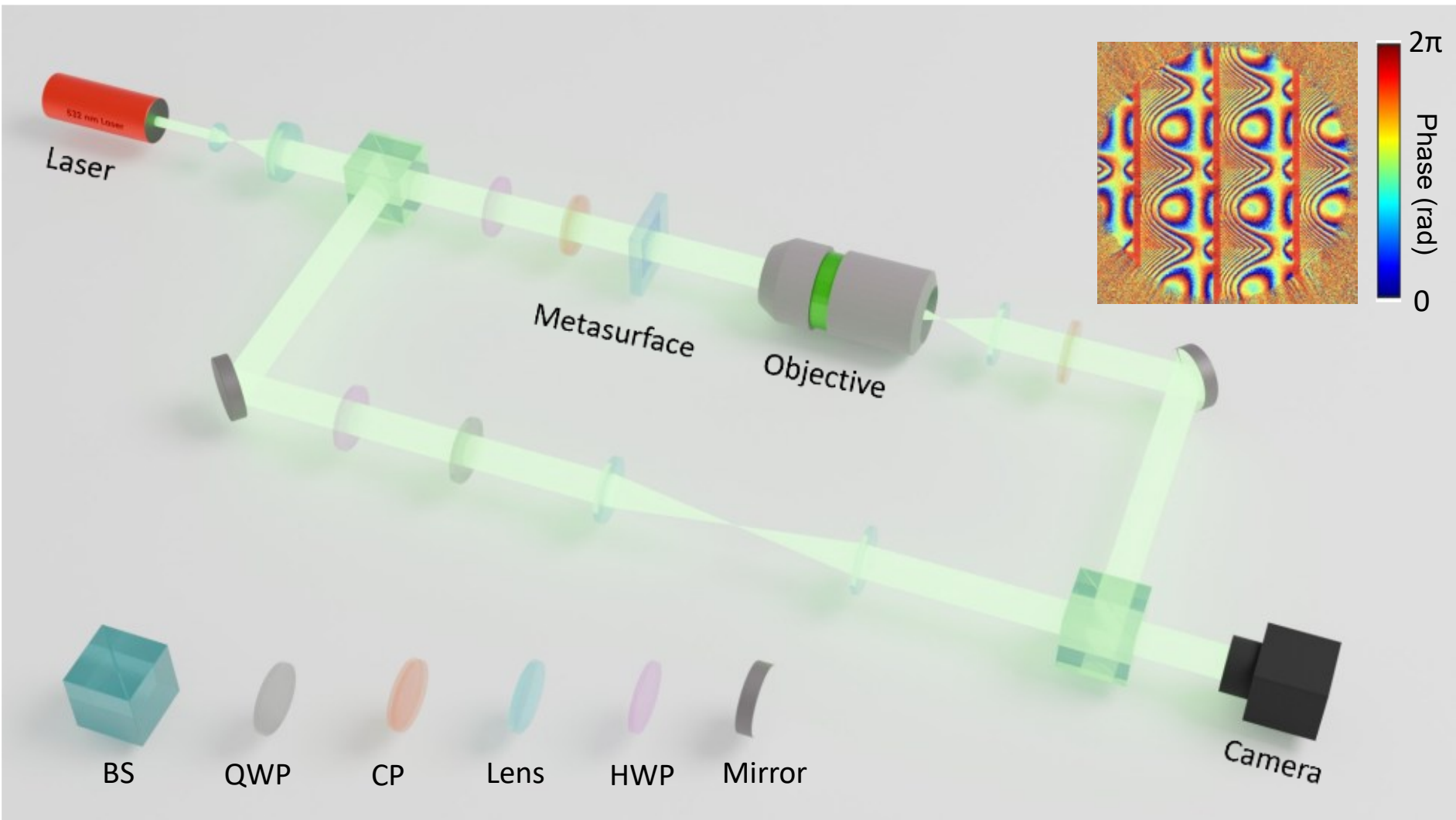


**Figure S2 Phase characterization setup.** A collimated laser beam with a wavelength of 532 nm passes through a beam splitter (BS) and splits into a reference beam and a test beam. The test beam is converted into circularly polarized light using a half-wave plate (HWP) and a circular polarizer (CP), and then directed onto the metasurface. A 20X objective lens is focused on the metasurface plane to collect the beam modulated by the nanostructures. A second BS recombines the reference beam with the test beam, which is tilted at an angle $\beta$, producing an interference pattern on the image sensor.

We also measured the phase profile of the metasurface using a commercial phase detection system (MetroLens, Ideaoptics). The schematic diagram of the optical system is shown in Fig. S2. The off-axis interference pattern $I$ can be described as[1]

$$I = I_1 + I_2 + \sqrt{I_1 I_2}\exp\left[i\left(\varphi + \Delta k \cdot x\right)\right] + \sqrt{I_1 I_2}\exp\left[-i\left(\varphi + \Delta k \cdot x\right)\right], \tag{S1}$$

where $I_1$ and $I_2$ denote the intensities of the test and reference beam, respectively, $\Delta k = k \cdot \sin\beta$ is the spatial frequency shift introduced by the angular tilt $\beta$ between the two beams, and $k$ is the wave number in vacuum. The Fourier transform of the interference pattern yields a central peak and two sidebands in the spatial frequency domain. These sidebands encode the phase information of the wavefront. The phase distribution can be retrieved by isolating one of the sidebands, applying an inverse Fourier transform to its complex amplitude, and extracting the phase angle. This process requires only a single image acquisition and does not rely on a phase-shifting technique.

It should be noted that the extracted phase information includes both the phase imparted by the metasurface and a background phase originating from the optical system. To account for this, a background measurement must be taken before inserting the metasurface. The background phase is then subtracted from the measured phase to obtain the final result. The corrected phase distribution is shown in the inset of Fig. S2.

## 3 Simulation and measurement of the focusing performance

The PSF measurement setup is shown in Fig. S3a. A collimated laser beam with a wavelength of 532 nm passes through a linear polarizer (LP) and a quarter-wave plate (QWP) to generate circularly polarized light. The metasurfaces are mounted on two motorized stages to enable controlled lateral displacement between them. Focal spots are captured using a microscope system, which can be translated along the optical axis. The three-dimensional intensity distribution of the focal region is then captured, as shown in Fig. S3b. We set $f_1$ = 3.4 mm, $f_2$ = 5 mm, $d_{max}$ = 69 μm, $l_g$ = 200 μm, $\lambda$ = 532 nm. According to Eqs. 1 and 2, the simulated focal length varies from 3.30 mm to 4.77 mm with the lateral displacement, in good agreement with the experimental results presented in Fig. 2f. Figure S3c shows both the simulated and measured focusing efficiency of the designed sub-metalens. The focusing efficiency is defined as the ratio of the optical power within the focused beam to the total incident optical power. The focused beam power is calculated by integrating the intensity within a region spanning twice the full width at half maximum (FWHM) centered at the focal points. The measured polarization conversion efficiency of the nanostructures is shown in Fig. S3d. The efficiency exceeds 60% across the visible spectrum, reaching up to 93% at the wavelength of 532 nm.

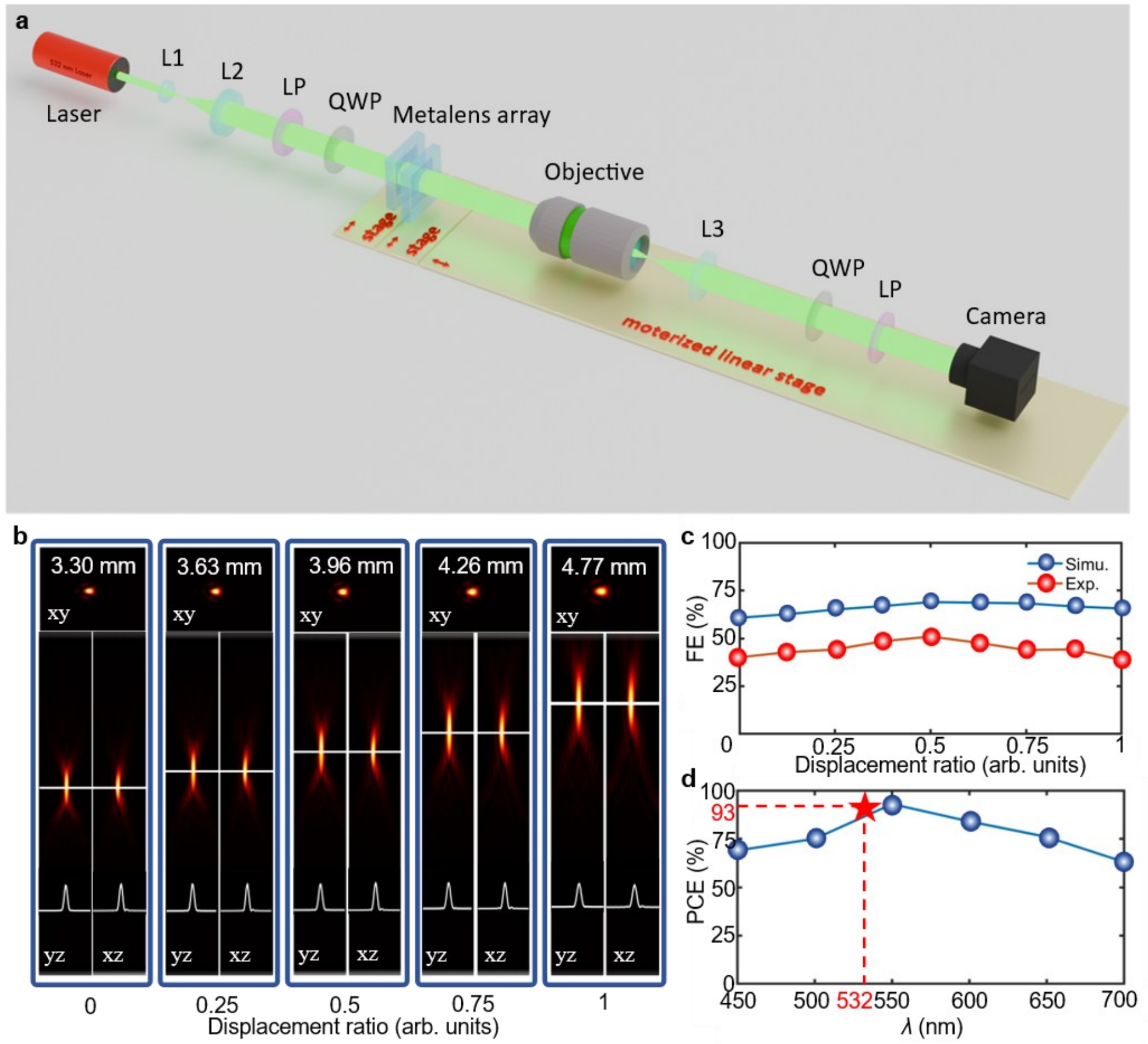


**Figure S3 Point spread function (PSF) measurement setup and focusing performance of the Alvarez metalens array (AMA). a** Schematic of the PSF measurement setup. **b** Intensity distribution of the focus spots in xy-, xz-, and yz-plane with different displacement ratios. Corresponding intensity profiles at the focal planes are shown below each distribution. **c** Focusing efficiency (FE) as a function of displacement ratio. **d** Measured polarization conversion efficiency (PCE) of the nanostructures.

In principle, the Alvarez mechanism relies on the near-field superposition of two phase profiles. The gap $l_g$ inevitably introduces diffraction-induced phase blurring and spatial crosstalk. Specifically, regions on the first metasurface with large phase gradients impart pronounced angular deflections to the incident rays. As these rays propagate across $l_g$, they deviate from their ideal trajectories before reaching the second metasurface.

To quantitatively assess this functional limit, the PSF and focusing efficiency across varying $l_g$ values are simulated, as shown in Fig. S4. At minimal gap distances, the focal spot closely resembles an ideal Airy disk. However, as $l_g$ increases, the sidelobes become increasingly prominent, and the focal spot elongates along the optical axis, as shown in Figs. S4a and S4b. While the focal length

changes slightly (<5%), the focusing efficiency notably decreases (>10%), as shown in Figs. S4c and S4d, respectively. In addition, our analysis demonstrates that reducing the numerical aperture (NA) effectively mitigates this degradation. A smaller NA requires a reduced phase gradient, which inherently suppresses the ray deflection angles and improves the system's tolerance to the gap $l_g$.

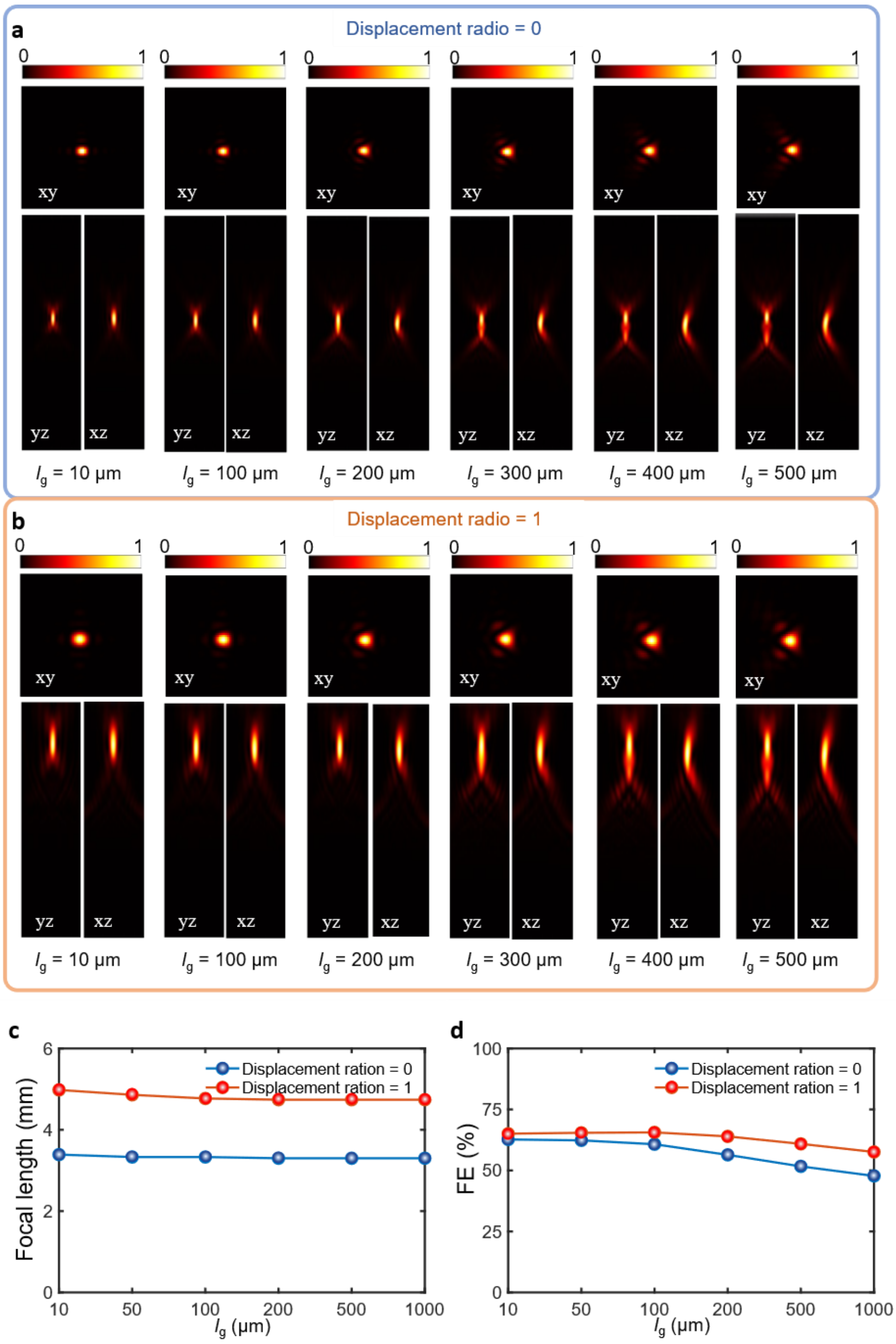


**Fig. S4 Simulated focusing performance of the Alvarez metalens array with different gap length $l_g$.** Simulated intensity distribution of the focus spots in xy-, xz-, and yz-plane when the displacement ratio is **a** 0 and **b** 1, respectively. **c** Focusing efficiency (FE) and **d** focal length vary versus $l_g$ with different displacement ratios.

The gap distance $l_g$ is fixed at 200 μm in our current configuration, which is primarily dictated by fabrication constraints. During the femtosecond laser direct writing process, the high peak intensity and subsequent rapid plasma expansion can cause surface ablation, microcracks, or localized material detachment if the structures are positioned too close to the air-glass interface. To ensure the uniformity of the nanostructures and preserve the optical quality of the surface, a safety margin of 100 μm below the wafer surface is strictly maintained. Consequently, assembling two such substrates inherently yields a minimum gap of $l_g = 200$ μm. While this gap guarantees structural stability, it could be further reduced in future iterations through post-process grinding and polishing to minimize propagation-induced phase distortion.

# II Design of an adaptive light-field imaging system

## 1. Assumption

For simplicity of computation, we make the following assumptions: (1) both the metalens and main lens are aberration-free and have fixed diameters; (2) the thickness of the metalens and main lens is neglected; (3) the unpatterned stripes between the sub-regions on the metasurfaces are neglected.

## 2. Symbol reference

**Table S1 Symbols used in the optical design of the plenoptic imaging system**

| Symbol | Definition |
|---|---|
| $T_L$ | Distance between object plane and image sensor |
| $l$ | Distance between the ideal focus position and the main lens |
| $A_L$ | Distance between object plane and the main lens |
| $B_L$ | Distance between main lens and image sensor |
| $B$ | Distance between the varifocal metalens array and image senor |
| $D_L$ | Diameter of the main lens |
| $D$ | Diameter of the sub-metalens |
| $f_L$ | Main lens focal length |
| $p$ | Pixel size of image sensor |

## 3. Parameter selection

In this section, we conduct an adaptive light-field imaging system (ALI) using commercially available optical elements. The designed system is capable of focusing on objects located 5 m away from the image sensor. The known parameters of the optical elements are listed in Table S2.

**Table S2 Known parameters of the used optical elements**

| Parameter | Value (mm) |
|---|---|
| $T_L$ | 5000 |
| $f_1, f_2$ | 3.33, 4.50 |
| $D_L$ | 12.76 |
| $D$ | 0.345 |
| $f_L$ | 100 |
| $p$ | 0.00345 |
| $\lambda$ | $5.32\times10^{-4}$ |

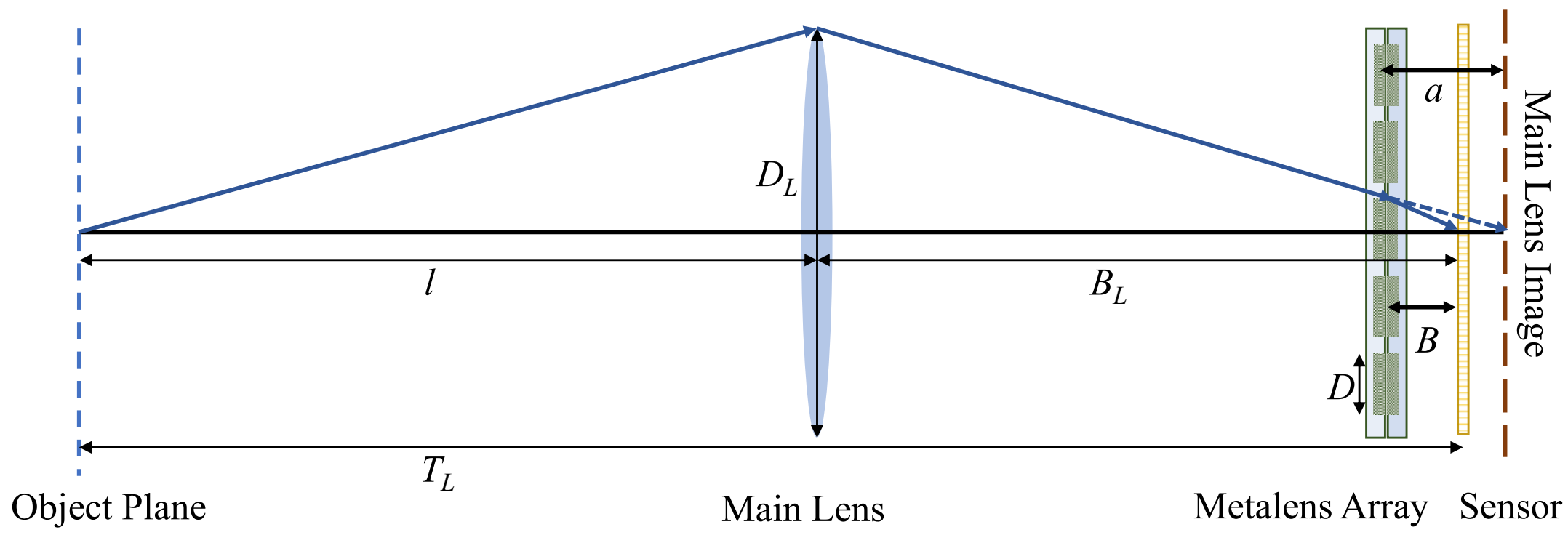


**Figure S5 Optical system of a plenoptic camera**

For a Galilean-type configuration, the image plane of the main lens is located behind the image sensor, as shown in Fig. S5. This implies that the effective resolution increases for distant objects and decreases for closer ones. However, a key advantage is that nearby objects appear larger in the sub-images, partially compensating for the reduction in resolution.

To fully utilize the image sensor, meaning the sub-images on the sensor are neither overlapping nor excessively separated, the working f-number of the main lens should match that of the metalens. Under this condition, the optimal distance between the metalens array and the image sensor can be calculated as[2]

$$B = \frac{DB_L}{D + D_L}. \tag{S2}$$

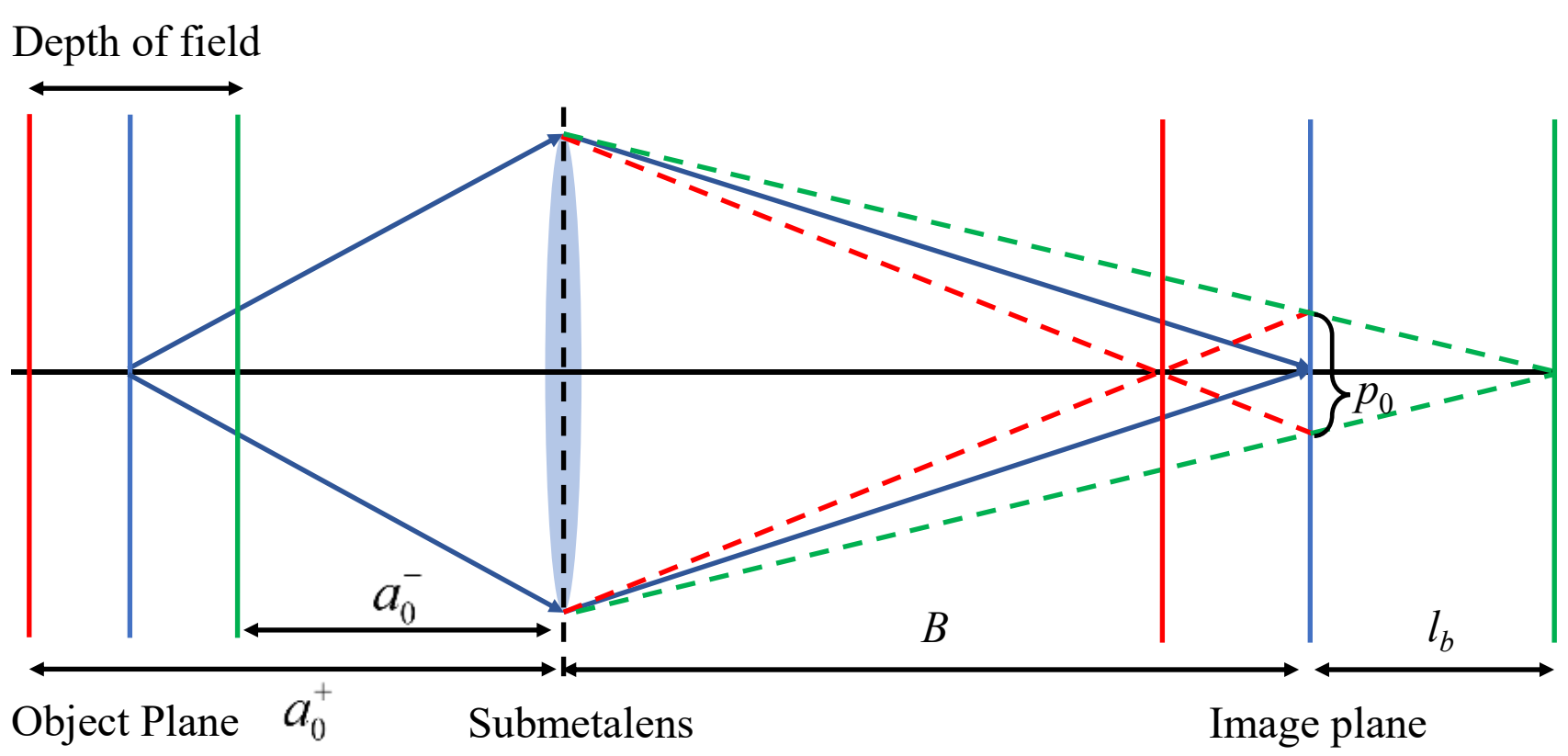


**Figure S6 Principle of the depth of field of sub-metalens**

The depth of field (DOF) of a sub-metalens refers to the axial range in object space over which a scene remains acceptably sharp on the image plane. It is closely related to the PSF of the sub-metalens, as shown in Fig. S6. The working PSF can be approximated by $p_\lambda = 1.22\lambda B/D$. The minimal resolvable spot size projected onto the image sensor $p_0 = \max\{p, p_\lambda\}$. Due to the finite size

of the working PSF, an object point located within a certain axial range around the nominal image plane produces a blur spot on the sensor that remains smaller than $p_0$, and is thus considered acceptably sharp[2]. For a beam originating from such an object point, this translates into an axial defocus tolerance $l_b$ on the image side of the focal plane, within which the blur spot does not exceed $p_0$. By applying the imaging relationships of the optical system, this permissible defocus range $l_b$ can be projected back into the object space, yielding the corresponding axial range where an object point can reside while still producing an acceptable focused image. This calculated range in object space defines the DOF $[a_0^-, a_0^+]$ based on the specified blur criterion. Using the triangular relationship $(B+l_b)/D=|l_b|/p_0$, and the lens conjugate equation $1/a_0+1/(B+l_b)=1/f$, the front and rear boundaries of the DOF can be calculated as

$$a_0^- = \left[\frac{1}{f_2}-\frac{1}{B}\left(1-\frac{p_0}{D}\right)\right]^{-1},\ a_0^+ = \left[\frac{1}{f_2}-\frac{1}{B}\left(1+\frac{p_0}{D}\right)\right]^{-1}. \tag{S3}$$

The object-side DOF of the metalens corresponds to the image-side DOF of the main lens. Therefore, the actual DOF in the object space of the overall system is primarily determined by the main lens. To fully exploit the DOF of the ALI in object space, the main lens should be placed closer to the image sensor. The optimal location of the main lens's focal plane coincides with the rear boundary of the ALI's DOF range ($a = B + l_b = |a_0^+|$). Under this condition, the distances between optical elements can be calculated using the following expression

$$\frac{1}{f_L}=\frac{1}{l}+\frac{1}{B_L+l_b},\ T_L = l + B_L. \tag{S4}$$

Using the kwon parameters listed in Table S2, the system parameters are calculated in Table S3.

**Table S3 Calculated parameters of the optical system**

| Parameter | Value (mm) |
|---|---|
| $B$ | 2.7 |
| $B_L$ | 98.3 |
| $l$ | 4901.7 |
| $p_0$ | $5.56\times10^{-3}$ |

# 3. Analysis of the resolution and the depth of field

The DOF of the designed ALI is determined by projecting the sub-metalens DOF into the object

space through the main lens. Accordingly, the actual front and rear boundaries of the DOF of the ALI, denoted as $b_0^-$ and $b_0^+$, can be expressed as

$$b_0^- = \left[\frac{1}{f_L} - \frac{1}{B_L - B + |a_0^-|}\right]^{-1}, \ b_0^+ = \left[\frac{1}{f_L} - \frac{1}{B_L - B + |a_0^+|}\right]^{-1}. \tag{S5}$$

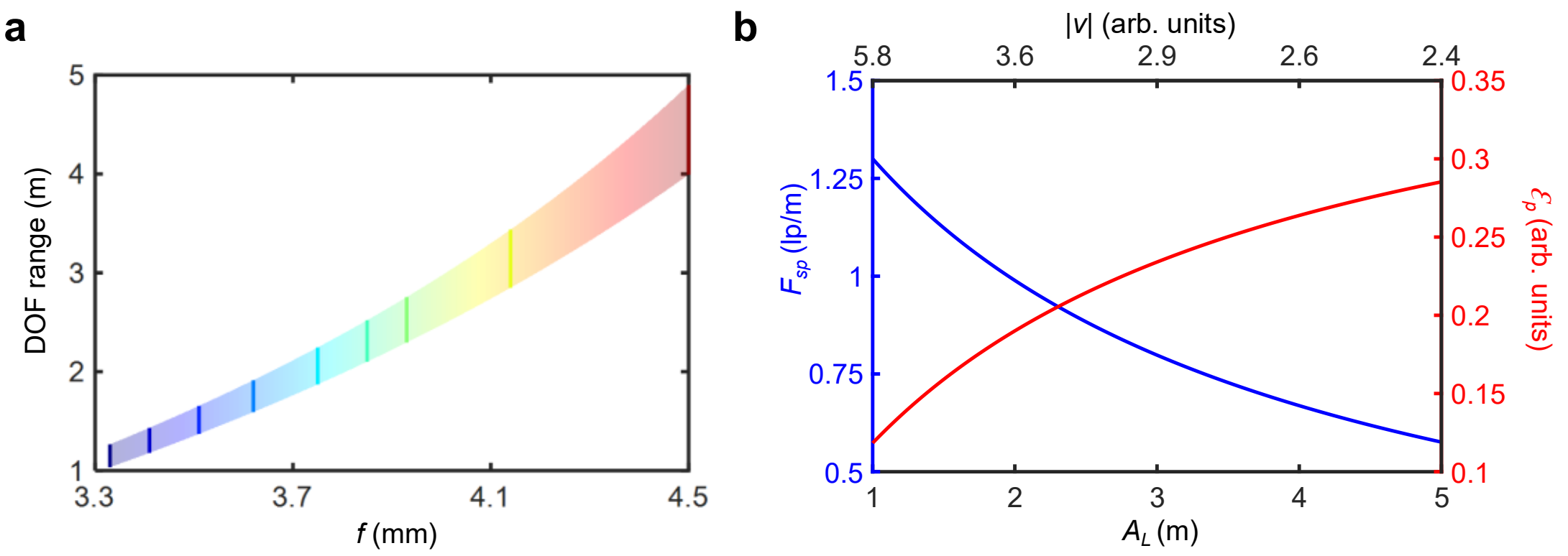


**Figure S7 Optical performance of the ALI. a** DOF range as a function of the sub-metalens focal length. **b** Object-space resolvable spatial frequency $F_{sp}$ (blue) and image-side effective resolution ratio $\varepsilon_P$ (red) vary with object distance $A_L$ and its corresponding angular sampling density $|v|$, respectively.

Since the focal length of the metalens array varies with lateral displacement, the DOF of the designed ALI can be tuned from approximately 1 m to 5 m, as shown in Fig. S7a. The image-side effective resolution ratio of an ALI, denoted as $\varepsilon_P$, is defined as the ratio between the system's resolution and the native resolution of the image sensor. It can be expressed as[2]

$$\varepsilon_p = \frac{1}{|v|} \frac{p}{\max\left\{\left|DB\left(\frac{1}{f} - \frac{1}{a}\right) - 1\right|, p_0\right\}}, \tag{S6}$$

where $|v| = |a/B|$ represents the angular sampling density, which quantifies how many metalenses simultaneously record the same point on the main-lens image plane. As shown in Fig. S7b, there is a trade-off between the spatial resolution ratio $\varepsilon_P$ and angular sampling density $|v|$ in this plenoptic configuration. Because the focal length of the designed metalens array $f$ can be continuously varied within a certain range, we can always achieve the minimal resolvable spot size on the image sensor. Then the effective resolution ratio can be written as

$$\varepsilon_p = \frac{1}{|v|} \frac{p}{p_0}. \tag{S7}$$

Equation S7 shows that the angular sampling density $|v|$ inevitably comes at the expense of the

effective spatial resolution ratio $\varepsilon_P$.

It is important to distinguish the image-side effective resolution ratio $\varepsilon_P$ from the object-space resolvable spatial frequency $F_{\mathrm{sp}}$, which is related to the resolution experiment in Fig. 4. With the magnification $M_L = (B_L\text{-}B+|a|)/A_L$, the object-space minimum resolvable feature size can be estimated as $\Delta_{obj} = p_0 / \varepsilon_p M_L$. The corresponding object-space resolvable spatial frequency is

$$F_{sp} = \frac{1}{2\Delta_{obj}} = \frac{\varepsilon_p M_L}{2p_0}. \tag{S8}$$

Equation S8 shows that the experimentally measured object-space resolution decreases for distant objects because the reduction in magnification $M_L$ dominates the trend, even though the image-side effective resolution ratio $\varepsilon_P$ may improve in the Galilean-type plenoptic configuration.

# 4. Principle of depth estimation

The depth of the recorded scene can be estimated from a single-shot image, particularly in regions with sufficient local contrast. By computing the sum of absolute differences of small pixel patches along the epipolar lines of sub-image pairs, the corresponding pixels across neighboring sub-images representing the same object point can be identified. These pixels typically occur in regions with distinct focal contrast, i.e., at edges, corners, or textured features. Once the depth values are determined for these pixels, the overall depth map of the scene can be reconstructed. As illustrated in Fig. S8a, the object depth corresponding to the metalens array can be calculated using the triangular relationship[3]

$$a\left(p_d\right) = \frac{BD}{2p_d} \tag{S9}$$

where $p_d$ = Disparity · $p$ denotes the parallax. The image point $a(p_d)$ on the sensor can be projected back through the main lens to the corresponding object point $l(p_d)$, according to the thin lens equation

$$l(p_d) = \left[\frac{1}{f_L} - \frac{1}{B_L - B + a(p_d)}\right]^{-1}. \tag{S10}$$

To analyze the achievable depth resolution with an ALI, it is important to note that the depth resolution depends on the distance from the metalens array. Specifically, the image-side depth resolution $\mathcal{E}_d$ of the sub-images improves with objects located farther from the system, and can be expressed as

$$\varepsilon_d = \partial_{p_d} l \Delta p = v^2 \frac{B}{D}\left(\frac{l - f_L}{f_L}\right)^2 \Delta p. \tag{S11}$$

The calculated depth resolution is plotted in Fig. S8b. When the object distance is close to 5 m, the depth resolution exceeds 0.5 m. This explains why the red nesting doll appears at a similar depth as the background board in the main text, making it difficult to distinguish between them in terms of depth.

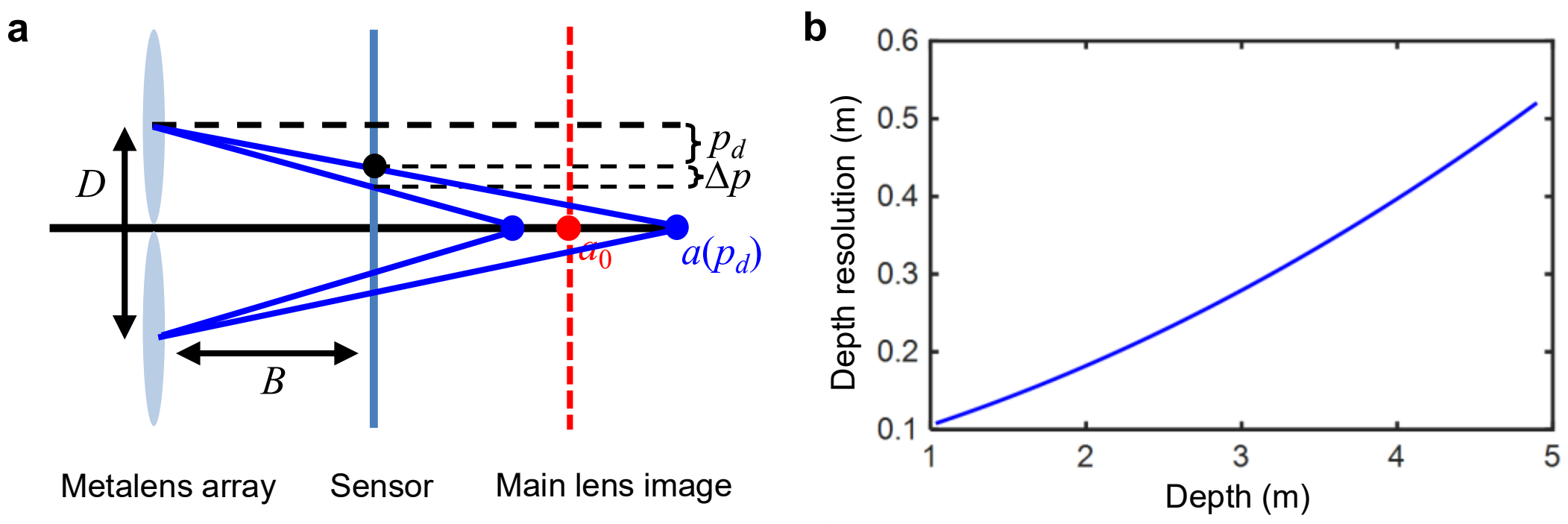


**Figure S8 Relationship between pixel resolution in sub-images and depth resolution. a** Within the plenoptic imaging framework, consider an image point located at a lateral displacement $p_d$ from the center of a sub-image. This point back-projects through the main lens and intersects the optical axis at point $a(p_d)$. For an adjacent pixel at an offset $p_d + \Delta p$, the corresponding back-projected ray intersects the optical axis at point $a(p_d + \Delta p)$. **b** Calculated depth resolution varies with depth.

## 5. Alignment and implementation

Aligning two metasurfaces posed significant challenges, particularly in achieving precise parallelism between the layers. The alignment procedure, illustrated in Fig. S2a, involved the following steps: (1) Focusing: A 50X magnification optical microscope objective is employed to sequentially focus on each metasurface layer by translating the motorized stage along the axial direction. A metasurface is considered in focus when the structural edges appear maximally sharp under visual inspection. (2) Parallelism verification: Parallelism between the two metasurfaces is confirmed when all boundaries within the field of view can be brought into sharp focus simultaneously by adjusting only the axial position ($z$-axis) of the objective lens without the need for independent tilt correction for either layer. (3) Lateral and angular alignment: The relative lateral and angular alignment of the two metasurfaces is assessed by observing the overlap of stripe regions

on each layer under the microscope. A condition of 0.5 lateral offset and zero relative rotation is defined by the complete overlap of these features. (4) Interlayer spacing validation: The axial travel distance of the motorized stage (~200 μm) required to shift focus from one metasurface to the other matched the designed interlayer spacing, validating the vertical separation between the two layers.

# III MultiLensFusion algorithm

## 1. Preprocessing

The preprocessing of light field white images involves the extraction of essential metadata --- such as lens positions and grid structure to facilitate subsequent image analysis. In the context of light field imaging, a white image refers to a calibration frame captured under uniform illumination across the entire metalens array. This image serves as a reference for identifying the spatial layout of the metalens array and is critical for later corrections and alignment processes. To enhance local structural features and suppress background illumination artifacts resulting from non-uniform lighting, a top-hat morphological operation is applied using an elliptical structuring element. This operation enhances local intensity variations, particularly around the metalens edges, thus facilitating robust segmentation. Thresholding is subsequently performed using Otsu's method, a widely used adaptive technique that determines the optimal threshold by maximizing the inter-class variance between foreground and background pixel intensities. The resulting binary image is refined through a border-clearing operation to remove connected components that touch the image boundary, as these are unlikely to represent valid metalens regions. In addition, small artifacts caused by illumination noise or sensor irregularities are removed to further clean the segmentation, ensuring that only prominent regions corresponding to the actual metalenses are preserved. The refined binary image is then labeled to identify individual connected components, with each component corresponding to a single metalens. To estimate the overall geometric structure of the array, the convex hull of the segmented regions is computed. The bounding box and the geometric properties of this convex hull are analyzed to estimate the grid layout, including the number of lenses in the horizontal and vertical directions ($N_x$ and $N_y$) and the spatial period $P$ of the metalens array.

The centroids of all detected metalens regions, representing the central coordinates of each metalens, are extracted and spatially sorted to align with the expected grid structure of the metalens array. These centroids are then reshaped into a two-dimensional array, corresponding to the presumed square layout of the array. However, due to uneven illumination or minor segmentation errors, the computed centroids may deviate slightly from the true center positions. Accurate localization of these centers is crucial for reliable disparity estimation in subsequent processing. Given the known square grid configuration of the metalens array, a geometric grid regularization

step is applied to correct distortions in the detected layout. This regularization minimizes the deviation between the segmented centroids and an ideal square grid by optimizing the grid's scale and orientation. The resulting correction ensures that the metalens positions conform closely to the intended geometric arrangement, thereby enhancing the accuracy of downstream computational tasks.

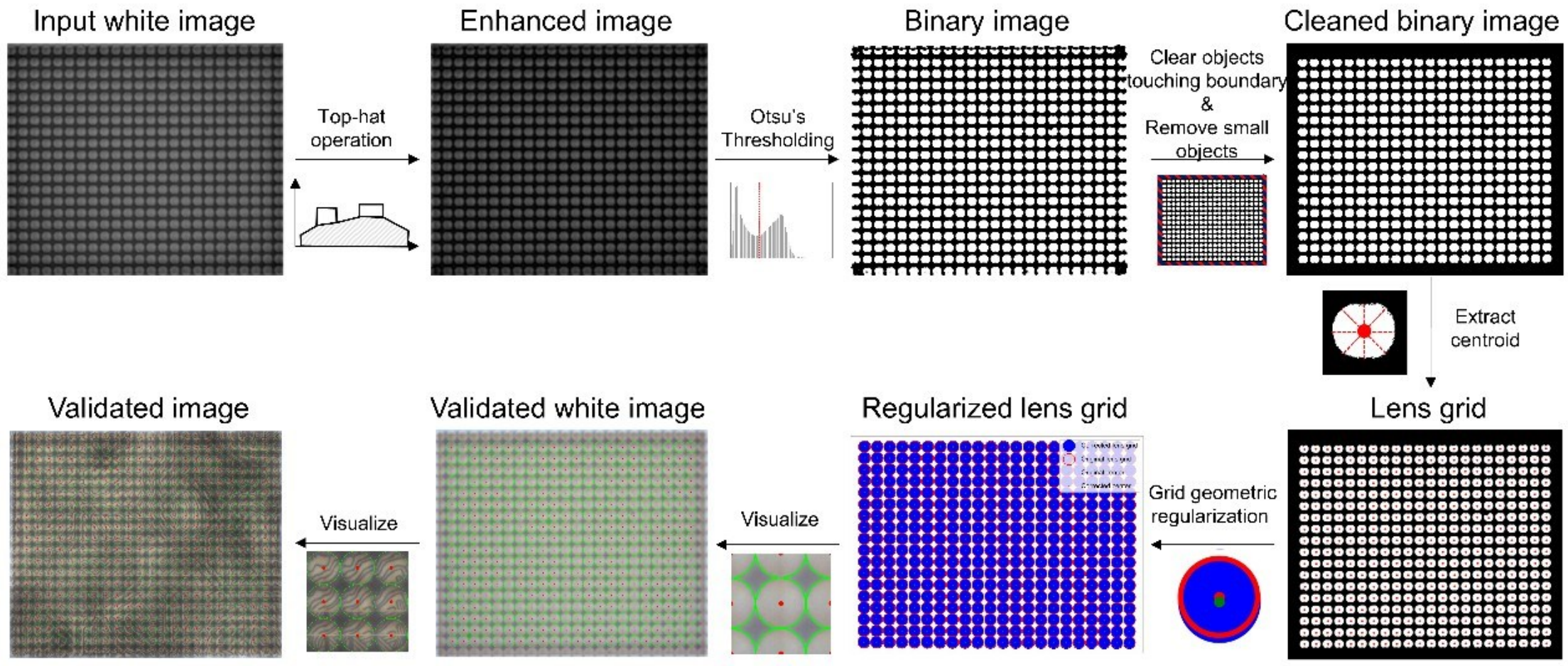


**Figure S9 Preprocessing pipeline for the white image, including feature enhancement, binary segmentation, centroid extraction, grid geometric regularization, and image visualization validation.**

As illustrated in Fig. S9, the corrected grid positions are visualized by overlaying them onto the original segmented grid. The initial lens grid is depicted using red hollow circles to indicate the metalens outlines and red dots to mark their centers. In contrast, the corrected grid is shown with blue solid circles and green dots representing the updated outlines and center positions. The zoomed-in view reveals subtle shifts between the original and corrected positions, underscoring the precision of the alignment refinement. This visualization effectively highlights the improvements achieved through the regularization process and confirms the enhanced conformity of the metalens array to its intended geometric structure. The corrected metalens positions are subsequently saved for use in calibration and later stages of light field image analysis. This preprocessing step ensures that deviations from the ideal grid caused by non-uniform illumination or segmentation artifacts are successfully mitigated, thereby establishing a robust foundation for accurate light field data processing.

## 2. Disparity calculation

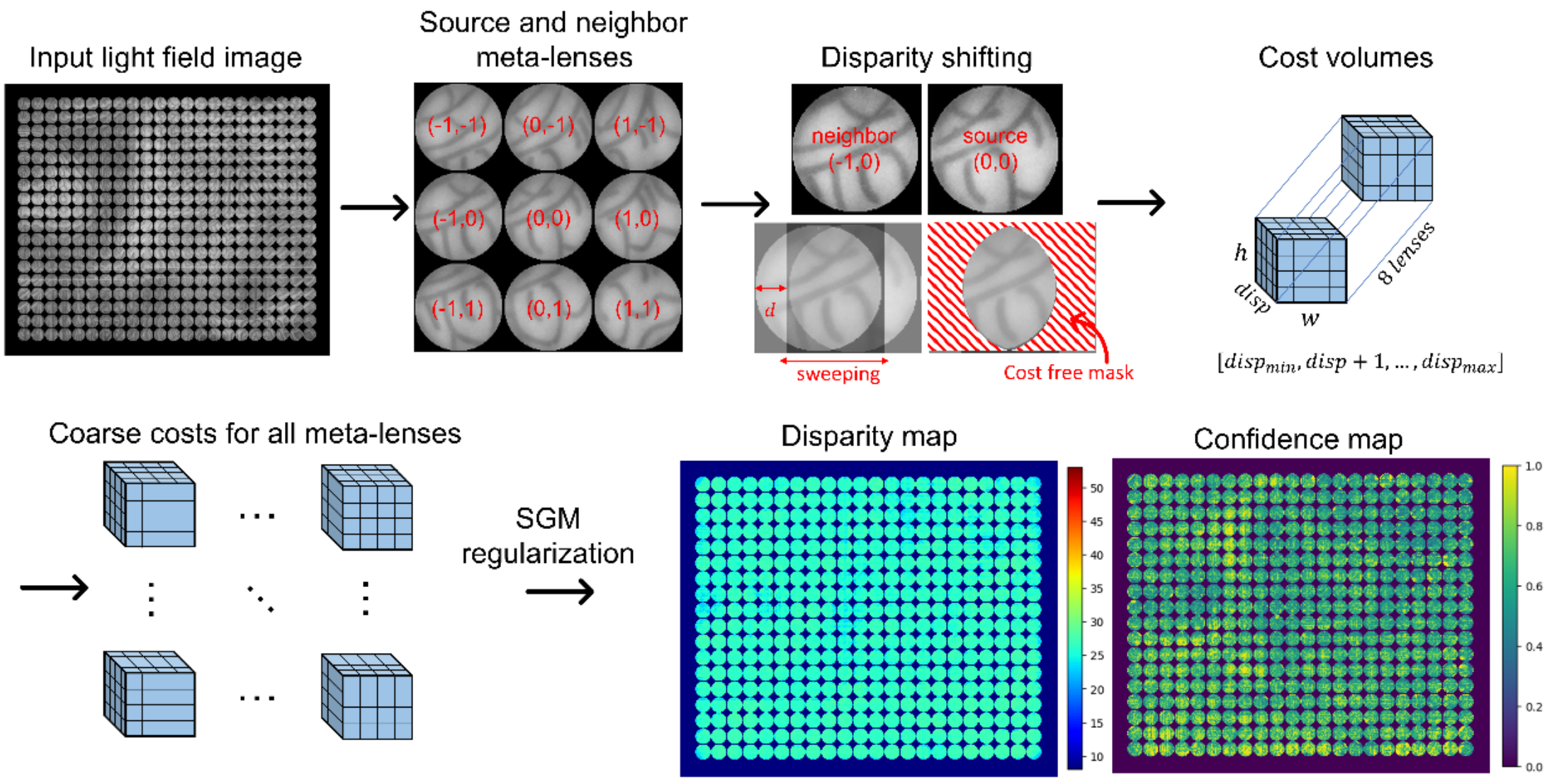


**Figure S10 Disparity calculation pipeline.** Each metalens is compared with its 8 neighboring metalenses to compute disparity costs across predefined disparity levels. A 4D cost volume is constructed using a plane-sweep approach, aligning the source metalens with shifted neighboring lenses. Matching costs are calculated, and cost-free masks are applied to exclude occluded or invalid regions. The disparity costs from all neighbors are merged and normalized into a single cost volume for each metalens. After applying Semi-Global Matching (SGM) for regularization, the final disparity maps and confidence maps for all metalenses are generated.

The spatial arrangement of the metalenses in the array follows a square grid, forming the structural basis of computation in the ALI. The depth estimation and digital refocusing capabilities of ALIs rely on capturing the angular and spatial variations of light rays as they pass through the metalens array. Unlike conventional imaging systems that record only light intensity, ALIs also encode directional information, enabling depth reconstruction through the analysis of disparities (shifts) between different views captured by neighboring metalenses. This principle enables the computational pipeline to synthesize images at different focal planes and to generate depth-aware representations of the scene.

To estimate depth, the algorithm computes matching costs over a predefined range of disparity levels for each sub-metalens, as illustrated in Fig. S10. This process involves selecting eight neighboring metalenses surrounding each central metalens according to a fixed spatial pattern based

on the square grid layout of the lens array. A key principle guiding this process is angular consistency, which refers to the alignment of light rays captured by adjacent metalenses. At the correct disparity level, rays from neighboring metalenses should correspond to the same scene point. By comparing pixel intensities across these neighboring views, the algorithm evaluates the degree to which each disparity level aligns with the actual scene geometry.

For each sub-metalens, the algorithm hierarchically computes disparity costs by comparing the source metalens with its neighboring metalenses across a predefined range of disparity levels. It begins with the construction of a 4D cost volume using a plane-sweep approach.[4-7] In this step, the source metalens image is aligned with shifted versions of its neighboring sub-images to obtain the potential disparity value. Matching costs are calculated based on the absolute intensity differences between the source and shifted neighboring images. To enhance accuracy, visibility masks are applied to exclude pixels that lie outside the valid aperture of the metalens radius or in occluded regions. This results in a 4D cost volume spanning neighboring metalenses, disparity levels, and spatial coordinates. The coarse disparity costs from all neighboring views are then aggregated and normalized into a unified cost volume for the source metalens. The merged cost volume incorporates disparity evidence from multiple viewpoints, promoting both spatial and angular consistency in the final depth estimation.

Once the cost volumes for the entire metalens array are constructed, a regularization step is applied to generate smooth and accurate disparity maps. Regularization is performed using a semi-global matching (SGM) algorithm, which balances local and global information by propagating disparity values across the image grid while penalizing abrupt depth transitions. This is achieved through the incorporation of smoothness constraints that discourage large disparity variations between adjacent pixels unless supported by strong image gradients, such as object edges. The SGM process ensures global consistency in the disparity map while preserving sharp depth discontinuities at structural boundaries.

Following regularization, disparity values are extracted by selecting, for each pixel, the disparity level corresponding to the minimum matching cost. To assess the reliability of these estimates, a confidence map is generated concurrently. Confidence values quantifying the trustworthiness of disparity estimation at each pixel are computed by comparing the minimum cost to the overall cost distribution across all disparity levels. The computation is carried out in the

logarithmic domain using a soft-min function, which effectively normalizes cost differences while maintaining numerical stability---particularly in cases where disparity costs exhibit large variations. The resulting confidence map highlights regions with reliable disparity estimates, enabling further refinement or selective processing in subsequent rendering stages.

## 3. Rendering

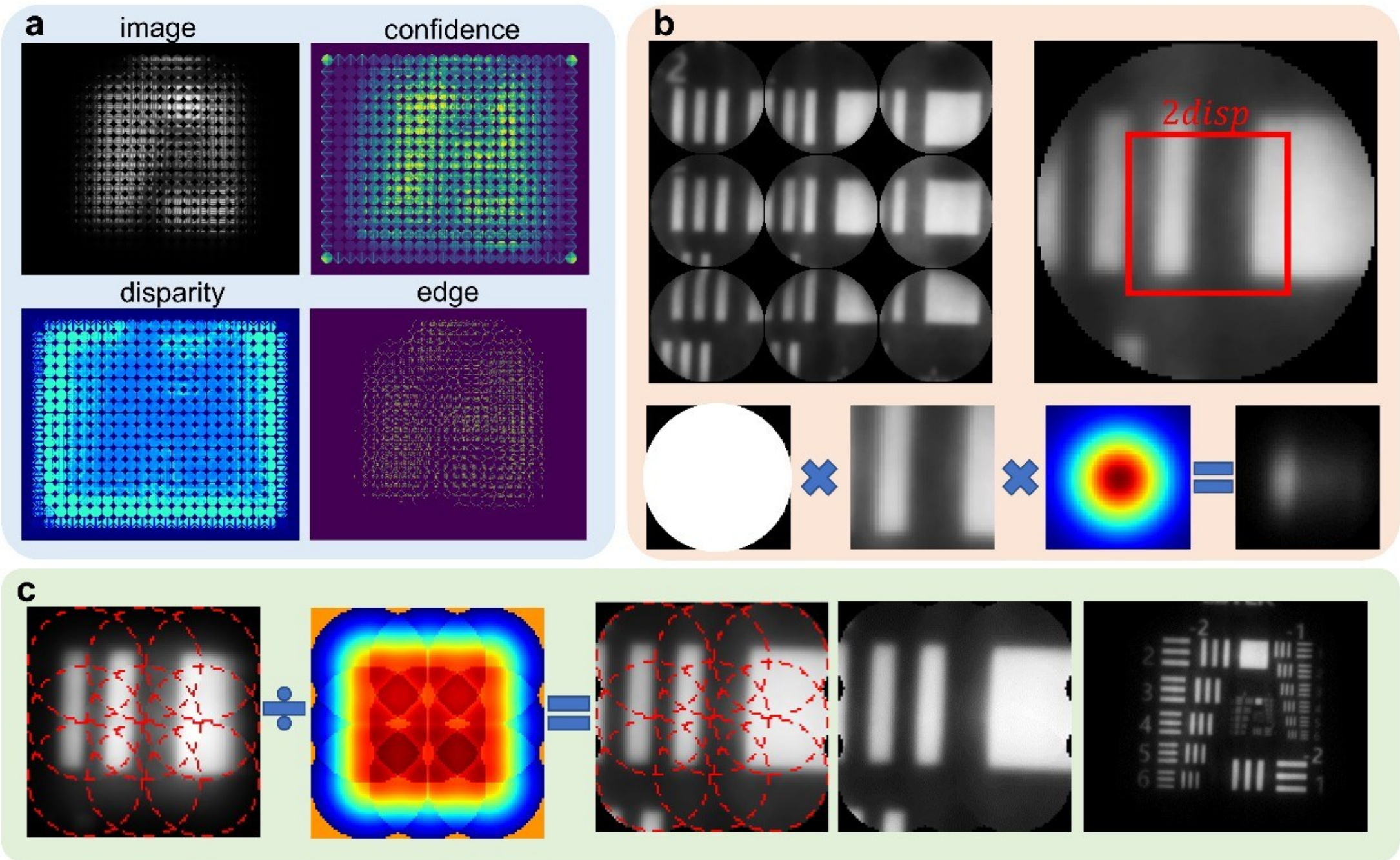


**Figure S11 Light field image rendering processing. a** Input multi-view image data, including raw image, disparity maps, confidence maps, and edge maps. **b** Rendering of a center metalens, which involves overlapping and stitching with its eight neighbor metalens. A square region, with a side length of $2disp$, is extracted from the metalens center. Circular mask and Gaussian weighting are applied to suppress the stitching boundary artifacts. **c** Final contributions from overlapping regions are balanced by normalizing with the accumulated Gaussian weights.

The rendering of light-field images entails the integration of multi-view image data, disparity maps, confidence maps, edge maps, and metalens calibration parameters to generate high-quality representations of a scene. This section presents a computational pipeline for light field image rendering, which enables accurate depth-aware image synthesis through patch-based sampling, interpolation, and multi-source disparity refinement techniques. The algorithm begins with the preprocessing of input data, which includes the raw light field image, a disparity map containing

depth information, an optional confidence map indicating the reliability of disparity estimates, and an edge map used to enhance depth estimation in regions with strong intensity gradients, as shown in Fig. S11a.

The spatial resolution of the final rendered image is determined by the calibration parameters of the metalens array, specifically the sub-metalens diameter and the positions of the sub-metalens center. Using these parameters, a local grid for the metalens array is constructed. Circular masks are applied to define valid sampling regions and to exclude edge artifacts introduced by sub-metalens boundaries. As a result, disparity, confidence, and edge data falling outside the circular mask are excluded from further processing. Figure S11b illustrates a simplified 3×3 metalens array, used to demonstrate the rendering process centered on the middle metalens.

The core of the rendering algorithm adopts a patch-based strategy, processing each metalens in the array individually. For each metalens, the regions of interest are extracted from the raw image, disparity map, and confidence map. Disparity estimation is refined by combining two complementary approaches: confidence-based disparity and edge-based disparity. The confidence-based disparity $d_{\text{conf}}$ is computed as the mean disparity of pixels whose confidence values exceed a predefined threshold, ensuring robust and reliable depth estimation.

$$d_{\text{conf}} = \begin{cases} \frac{1}{N_{\text{conf}}} \sum_{j \in P_{\text{conf}}} d_j, & N_{\text{conf}} \geq 10 \\ 0.1, & \text{otherwise} \end{cases} \tag{S12}$$

where $P_{\text{conf}}$ denotes the set of pixels in the metalens with confidence $\text{conf}_j$ exceeds a predefined threshold $T_{\text{conf}} = 0.3$. The total number of pixels in $P_{\text{conf}}$ is denoted by $N_{\text{conf}}$, and $d_j$ is the disparity at pixel $j$.

$$d_{\text{edge}} = \begin{cases} \frac{1}{N_{\text{edge}}} \sum_{j \in P_{\text{edge}}} d_j, & N_{\text{edge}} \geq 10 \\ 0.1, & \text{otherwise} \end{cases} \tag{S13}$$

At the same time, the edge-based disparity $\text{d}_{\text{edge}}$ is calculated from high-gradient regions in the edge map to capture fine structural details of the scene. Define $P_{\text{edge}}$ as the set of edge pixels in the metalens, and $N_{\text{edge}}$ as the total number of pixels in $P_{\text{edge}}$. To balance the contributions of $d_{\text{conf}}$ and $d_{\text{edge}}$, the absolute difference $\Delta d = | d_{\text{conf}} - d_{\text{edge}} |$ is computed and normalized to adjust their weights dynamically.

$$n_{\Delta d} = \frac{\max\left\{\left|d_{\text{conf}} - d_{\text{edge}}\right|,\ \Delta d_{\max}/2\right\}}{\Delta d_{\max}/2}, \tag{S14}$$

where $\Delta d_{max} = d_{max} - d_{min}$ is the disparity range. The final disparity $disp$ for each metalens is obtained as a weighted combination of $d_{conf}$ and $d_{edge}$, with dynamic weight adjustment based on the disparity difference $n_{\Delta d}$ to balance the contribution of the two sources.

$$\text{disp} = (c_1 - c_2 n_{\Delta d}) d_{\text{conf}} + (c_1 + c_2 n_{\Delta d}) d_{\text{edge}}, \tag{S15}$$

where $c_1 = 0.5$ is the balance coefficient, $c_2 = 0.3$ is the refinement ratio coefficient. When the difference between $d_{conf}$ and $d_{edge}$ is large ($|d_{conf} - d_{edge}| > \Delta d_{max}/2$), the contribution of $d_{edge}$ increases with higher reliability. Conversely, when $d_{conf}$ and $d_{edge}$ are similar, the final disparity simplifies to disp $\approx d_{conf} \approx d_{edge}$. After determining the final disparity, it is used to guide the sampling and interpolation within the corresponding metalens patch. A square region centered at the metalens is extracted, with a side length defined as $2 \cdot \text{disp}$. This region is then resampled to the target resolution for seamless stitching with neighboring patches. As shown in Fig. S11c, final contributions from overlapping regions are balanced by normalizing with the accumulated Gaussian weights. This normalization enforces consistent blending among adjacent metalens patches, thereby reducing edge discontinuities and improving the fidelity of the final stitched reconstruction.

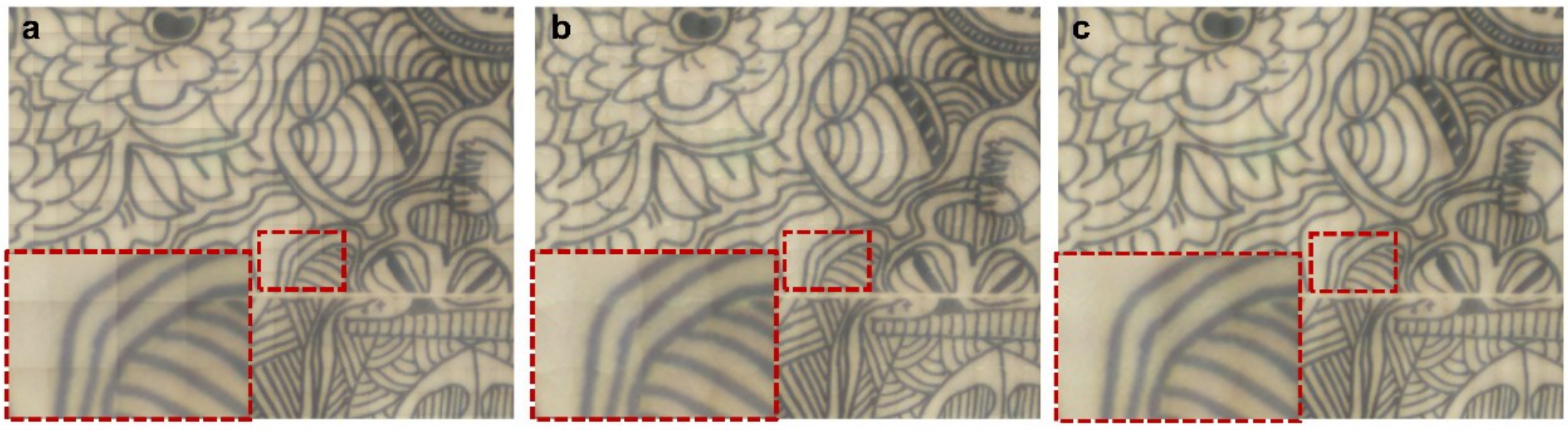


**Figure S12 Comparison among stitching strategies.** **a** square patch, **b** circular patches, and **c** circular patches with Gaussian weighting.

The shape of each metalens patch plays a critical role in minimizing stitching artifacts and ensuring seamless blending in the rendered image. As illustrated in Fig. S12a, square patches often introduce visible artifacts along their boundaries due to abrupt intensity transitions between adjacent patches. In contrast, circular patches help reduce such artifacts by restricting sampling to areas that align more naturally with the metalens geometry, as demonstrated in Fig. S12b. This improvement results from the fact that circular edges avoid the sharp discontinuities characteristic of square patches and more closely match the natural intensity falloff at the edges of the metalens apertures, as governed by the cosine-fourth-power law. To further suppress stitching artifacts and enhance

continuity between adjacent patches, a Gaussian weighting mask is applied to the sampled data. The mask attenuates the contributions of pixels near the patch boundaries, promoting smoother transitions and effectively reducing edge-related artifacts, as illustrated in Fig. S12c.

During rendering, the image extracted from the central metalens overlaps with those from its surrounding metalenses, as indicated by the red dashed circles in Fig. S12c. The final rendered image and disparity map are normalized by dividing the accumulated values by the corresponding Gaussian weight maps, thereby balancing the contributions from overlapping regions and ensuring consistent intensity across the entire field of view.

In addition, we find that the chromatic aberration in our imaging system does not severely degrade the overall image quality even before any rendering or stitching process. In our system, the achromatic main lens first forms a color-corrected intermediate image of the scene. The AMA then acts on this intermediate image as a local sampling and relay element over a short propagation distance, and each sub-metalens operates in a highly paraxial regime. Since each sub-metalens captures only a small local portion of the intermediate image, the effective field angle is small. As a result, the chromatic error introduced by the AMA is dominated by longitudinal chromatic aberration, rather than strong transverse chromatic aberration. This greatly suppresses visible lateral color fringing. Furthermore, the NA of the sub-metalenses is only 0.038-0.052. Such a small NA gives a large depth of focus ($\sim\lambda/NA^2$), and also reduces the sensor-plane blur caused by chromatic focal shift. Therefore, although different wavelengths focus at different axial positions, the corresponding wavelength-dependent blur on the sensor remains modest.

## 4. Merge all-in-focus images

To achieve an all-in-focus image with a wide depth of field by merging photos captured by the ALI with different focal lengths, the proposed algorithm leverages sharpness evaluation and metalens-based selection to integrate data from metalenses of small, medium, and large focal lengths. This MultiLensFusion process ensures that the sharpest regions from each focal length are preserved, resulting in a single composite image with enhanced depth perception and clarity across all depth planes.

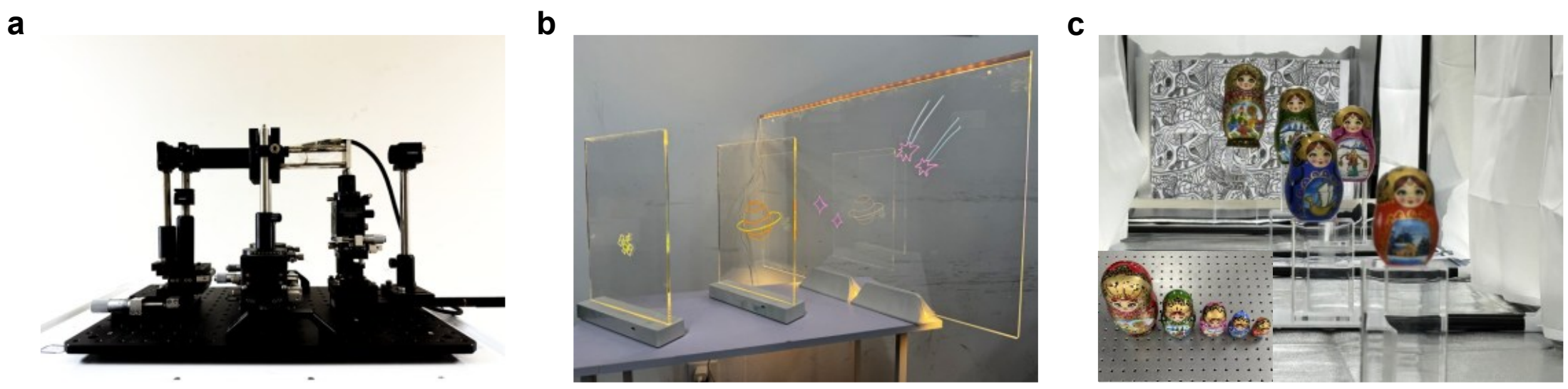

**Figure S13 The objects and setup used in all-in-focus imaging. a** Implemented optical system. **b** Light boards with two-dimensional patterns. **c** Nesting dolls placed in the photo studio.

The implemented optical system is shown in Fig. S13a, and the objects used for all-in-focus imaging are shown in Fig. S13b and S13c. The pipeline begins by loading the images captured by the ALI using metalenses with different focal lengths, as shown in Fig. S14a, along with their corresponding metadata. The metadata for each metalens includes its original image, disparity, depth, confidence, edge, center pixel coordinates, and metalens array coordinates, all of which are stored in a dictionary structure. Each sub-metalens is treated as an independent entity within this dictionary, enabling flexible handling of overlapping regions across different focal lengths. To evaluate image clarity, the sharpness of each sub-metalens image is computed using the Laplacian operator, where the variance of the Laplacian serves as a quantitative sharpness metric. This metric is used to identify, at each spatial location, the focal length (short, medium, or long) that yields the sharpest and most focused image of the scene. To construct the final all-in-focus metalens array, the algorithm initializes an empty dictionary to store the sharpest metalens data for each spatial position. It then iterates through the sets of metalens images captured at different focal lengths. For each position, the algorithm compares the sharpness of the candidate images and updates the dictionary if a metalens of a different focal length provides a sharper result. This iterative selection process ensures that the dictionary retains the metalens yielding the highest image sharpness for each location, thereby producing an all-in-focus composite image that integrates the most focused regions across all focal lengths.

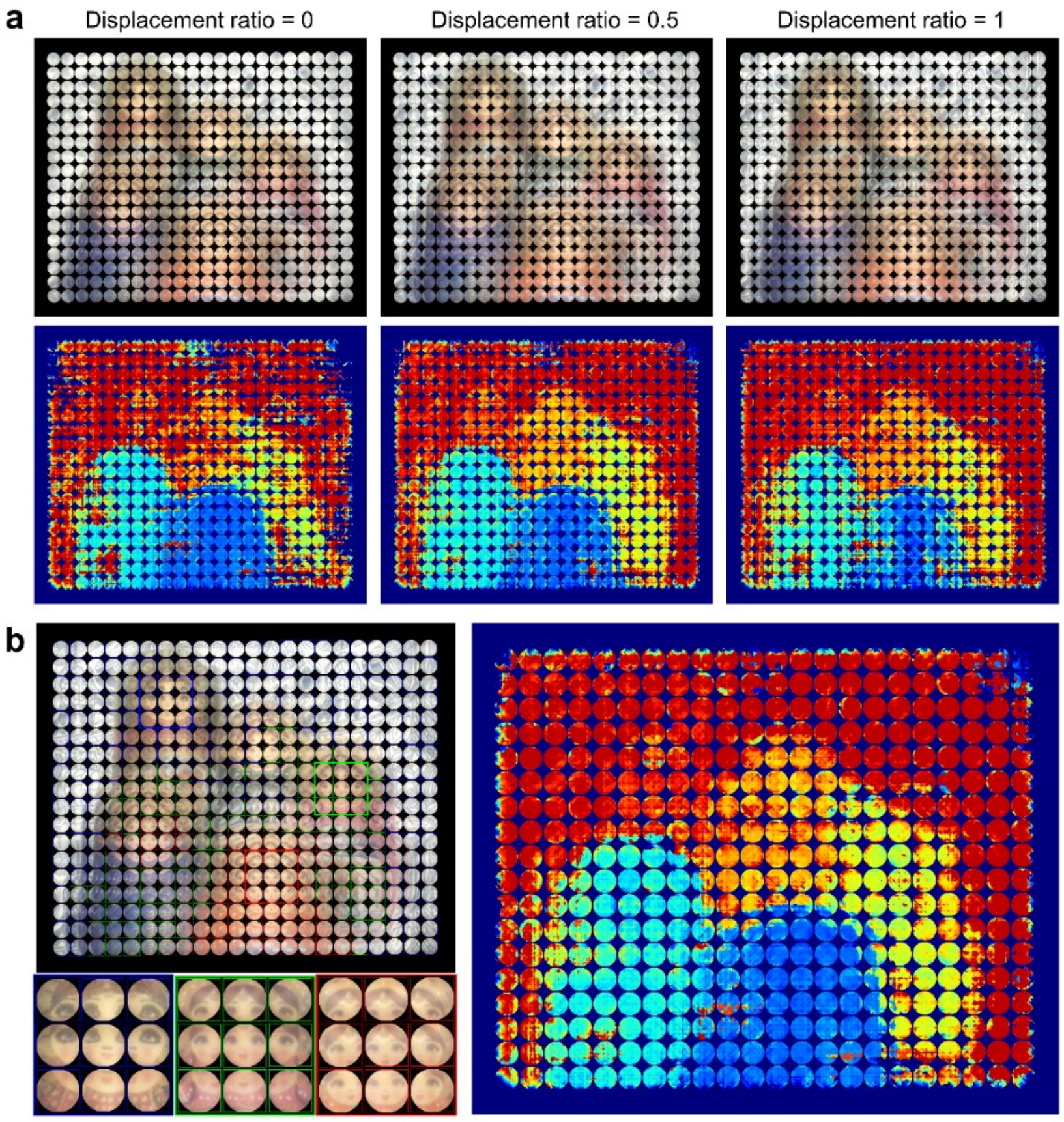


**Figure S14 Merging the all-in-focus light field image. a** Raw light-field images captured at three displacement ratios: 0, 0.5, and 1, along with their corresponding disparity distributions. **b** Merged all-in-focus light field image, where each sub-image (corresponding to a metalens) is selected as the sharpest one from the three focal length source images. Sub-images are outlined with different colors based on their source displacement ratio: blue for 0, green for 0.5, and red for 1. The bottom-left panel shows zoomed-in sub-images from different focal length sources. The right panel displays the updated all-in-focus disparity map, where each sub-map corresponds to the selected metalens source.

Once the sharpest images are selected, they are spatially aligned and rendered into a global image coordinate frame. Each metalens image is integrated into the composite image using its associated metadata. Fig. S14a shows the raw images and disparity maps corresponding to the three focal lengths. In the merged all-in-focus metalens array dictionary, illustrated in Fig. S14b, the contribution of each metalens is governed by its focal length and the depth of the corresponding object. To visualize the contributions from metalenses of different focal lengths, the boundaries of their valid regions are color-coded: red indicates short focal lengths, green represents medium focal lengths, and blue corresponds to large focal lengths. This color-coding provides an intuitive

representation of how various focal lengths contribute to different depth layers of the final image. The final composite image is generated by blending the selected sharp images from each focal length into a unified output. This fused image exhibits an extended depth of field, maintaining visual sharpness across a broad range of scene depths. By integrating depth-dependent sharpness information from multiple focal planes, the resulting image achieves a consistent focus throughout the entire scene.

# Reference


1. Zhao, M., Chen, M. K., Zhuang, Z. P., et al. Phase characterisation of metalenses. *Light Sci. Appl.*, **10**, 52 (2021).
2. Perwass, C., Wietzke, L. Single lens 3d-camera with extended depth-of-field. *Proc Spie*, **8291**, 829108 (2012).
3. Liu, X., Chen, M. K., Chu, C. H., et al. Underwater binocular meta-lens. *ACS Photonics*, **10**, 2382-2389 (2023).
4. Jeon, H.-G., Park, J., Choe, G., et al. Accurate depth map estimation from a lenslet light field camera. *2015 IEEE Conference on Computer Vision and Pattern Recognition (CVPR)*. 1547-1555 (2015).
5. Kalantari, N. K., Wang, T.-C., Ramamoorthi, R. Learning-based view synthesis for light field cameras. *ACM Trans. Graphics*, **35**, 1-10 (2016).
6. Palmieri, L., Koch, R., Veld, R. O. H. The plenoptic 2.0 toolbox: Benchmarking of depth estimation methods for mla-based focused plenoptic cameras, *proceedings of he 2018 25th IEEE International Conference on Image Processing (ICIP)*, F, 2018. IEEE, Published (Year).
7. Wanner, S., Goldluecke, B. Globally consistent depth labeling of 4d light fields. *2012 IEEE Conference on Computer Vision and Pattern Recognition*. 41-48 (2012).
