# Supplementary material for: Varifocal Alvarez metalens array for adaptive light-field imaging
Source: Nat Commun. 2026 Jun 23;17:7882. doi: 10.1038/s41467-026-74606-8 (PMC13442863; doi:10.1038/s41467-026-74606-8)
Supplement: Supplementary file 2 [file 41467_2026_74606_MOESM2_ESM.pdf]

Description of Additional Supplementary Files

Supplementary Movie 1: Change in the focal spot with lateral displacement.
