# Supplementary material for: Varifocal Alvarez metalens array for adaptive light-field imaging
Source: Nat Commun. 2026 Jun 23;17:7882. doi: 10.1038/s41467-026-74606-8 (PMC13442863; doi:10.1038/s41467-026-74606-8)
Supplement: Supplementary file 3 [file 41467_2026_74606_MOESM4_ESM.pdf]

nature portfolio

Peer Review File

Varifocal Alvarez metalens array for adaptive light-field imaging

Corresponding Author: Professor Din Ping Tsai

Parts of this Peer Review File have been redacted as indicated to remove third-party material.

This file contains all reviewer reports in order by version, followed by all author rebuttals in order by version.

Version 0:

Reviewer comments:

Reviewer #1

(Remarks to the Author)
This manuscript describes a pioneering and elegantly designed varifocal Alvarez metalens array (AMA) that effectively bridges the gap between flat optics and computational imaging. By integrating a tunable metasurface array into a plenoptic architecture, the authors have achieved a significant milestone in dynamic optical adaptability. The demonstration of continuous focal length tuning via simple lateral displacement, coupled with the sophisticated MultiLensFusion algorithm , represents a robust and innovative approach to "all-in-focus" 3D scene reconstruction. The system-level implementation is impressive and well-executed, offering a compelling vision for next-generation light-field platforms. While the conceptual framework is strong, several technical aspects regarding characterization and physical modeling require further clarification to ensure the manuscript meets the high standards of rigor for publication.
1. In the resolution experiments, the resolvable spatial frequency decreases significantly for distant objects, which is consistent with the system geometry and angular sampling constraints. The manuscript would benefit from a more explicit quantitative analysis of the trade-off between spatial resolution and angular sampling in the plenoptic configuration. Providing theoretical limits or analytical estimates would improve the completeness and rigor of this evaluation.
2. In the Introduction, the manuscript groups Refs. 34-43 under MEMS-based tunable metalenses. However, only Ref. 34 and Ref. 39 employ genuine MEMS actuation. The remaining references rely on external mechanical translation or non-MEMS mechanisms. This classification is therefore not entirely accurate.
3. The authors state that the metasurface patterns are inscribed approximately 100 μm beneath the wafer surface to provide subsurface protection. However, the high-magnification SEM images shown in Fig. 1e and Fig. S1e provide extremely clear details of the nanostructures. Given that standard SEM imaging is a surface-sensitive technique and cannot penetrate a 100 μm silica layer, the manuscript should clarify how these SEM images were obtained. Specifically, the authors should indicate whether the images were acquired from a cross-sectioned sample or from a dedicated surface-patterned test wafer.
4. The authors state that "The metasurfaces are embedded within one-inch-diameter silicon wafers." However, throughout the rest of the manuscript and the "Methods" section, the substrate is referred to as fused silica. Since silicon is opaque at the operating wavelength (532 nm) and the laser-writing process described is specifically for transparent dielectrics, this appears to be a significant typographical error.
5. The authors specify that the total propagation distance between the two layers lg is about 200 μm. While the Rayleigh-Sommerfeld diffraction method is used to compensate for this separation, the manuscript does not provide a formal justification for selecting this specific depth. The authors are encouraged to clarify the rationale behind the choice of 200 μm, including whether it is determined by fabrication constraints, mechanical considerations, or optical performance optimization. In principle, the Alvarez mechanism relies on the near-field superposition of two phase profiles. As lg increases, diffraction may introduce phase blurring and spatial crosstalk between the 345 μm pitch sub-lens regions. A quantitative discussion or simulation addressing the functional limit of lg, and its impact on PSF quality or focal length accuracy, would significantly strengthen the physical justification of the design.
6. The proposed AMA does not incorporate an achromatic design. Theoretically, a metasurface with a fixed phase profile should exhibit significant chromatic aberration across the visible spectrum. However, the imaging results presented in the manuscript (especially the full-color scenes in Fig. 4 and Fig. 5) do not show severe color fringing or dispersion-induced blurring. Please provide a explanation for this observation.
7. In Fig. 1a, the schematic of the 2×2 AMA with tunable focus spots requires improvement. The phase pattern illustrated on the metasurface does not clearly resemble an Alvarez-type cubic phase profile, which may cause confusion regarding the

underlying operating principle. In addition, the red curves shown in the schematic are not explained in the figure caption or main text, and their physical meaning (e.g., focal shift, beam propagation, or displacement direction) remains unclear.
8. The manuscript states that the metasurface structures are fabricated using femtosecond laser direct writing inside the substrate; however, the fabrication parameters are not described in sufficient detail. For clarity and reproducibility, the authors should provide the complete laser processing parameters, including laser wavelength, pulse duration, repetition rate, objective numerical aperture (NA), effective voxel size (lateral and axial), and the minimum achievable feature size. In addition, it is important to clarify how the minimum writable feature size and structural pitch compare with the operating wavelength. To achieve effective phase modulation without generating unwanted higher-order diffraction, the nanostructure period must satisfy the subwavelength condition (i.e., smaller than the working wavelength). The manuscript should therefore specify the structural pitch and provide a quantitative comparison between feature size, pitch, and operating wavelength, together with a discussion of phase sampling requirements and discretization error.
9. In Figure S3, the caption and accompanying description appear incomplete. Specifically, Fig. S3b is not described or discussed in the text. The Supplementary Information should be revised to ensure that all subfigures, including Fig. S3b, are properly described, clearly labeled, and consistently referenced. This would improve clarity and avoid potential ambiguity.

Reviewer #2

(Remarks to the Author)
This manuscript presents a compelling demonstration of a varifocal Alvarez metalens array (AMA) integrated into a plenoptic imaging system. The concept of achieving dynamic optical adaptability through lateral displacement is innovative, and the combination with a MultiLensFusion algorithm to achieve an extended depth-of-field is significant. The work is well-structured and falls within the scope of the journal. However, several critical points need to be addressed before publication.
1. The introduction provides a good overview but omits key references highly relevant to the work. The authors should discuss and cite recent advances in "Polarization-separating Alvarez metalenses" and "Large Area Tunable Alvarez Metalenses via Stepper Photolithography." Including these will better position the novelty of this work.
2.The author mentioned in lines 116 and 305 that the metasurface was fabricated from silicon and fused silica respectively, which is contradictory. It is suggested to modify the expression. Besides, the metasurface pattern is embedded approximately 100 micrometers below the sample surface. How was the SEM image captured?
3. The relationship between the physical dimensions of the nanostructures shown in the SEM image and the achieved phase profile is not explained, What is the morphology of the micro-nano structure in the depth direction?
4. The manuscript mentions laser writing, but the structures in Fig. 1e appear to have sub-100 nm features, which is challenging for standard laser lithography. How these high-resolution structures were fabricated and imaged. How to control the fabrication accuracy of the structure and optimize its working efficiency?
5.The manuscript notes a "slight deviation" between the simulated total phase and the ideal phase profile. A detailed explanation of the source of this deviation is necessary to validate the design methodology and assess its limitations.
6. The reconstructed "all-in-focus" images in Figure 5 are presented without any scale information.
7.This paper demonstrates the imaging capabilities of the Alvarez superlens array and how to further enhance the imaging resolution. What are the main limiting factors?

Version 1:

Reviewer comments:

Reviewer #1

(Remarks to the Author)
All my concerns have been satisfactorily addressed, and I find the manuscript acceptable for publication in its current form.

(Remarks on code availability)

Reviewer #2

(Remarks to the Author)
I am satisfied with the authors' responses and the modifications made to the manuscript and Supplementary Information. All my previous points have been adequately resolved. I recommend publication in its current form.

(Remarks on code availability)

Open Access This Peer Review File is licensed under a Creative Commons Attribution 4.0 International License, which permits use, sharing, adaptation, distribution and reproduction in any medium or format, as long as you give appropriate credit to the original author(s) and the source, provide a link to the Creative Commons license, and indicate if changes were made.
In cases where reviewers are anonymous, credit should be given to 'Anonymous Referee' and the source.
The images or other third party material in this Peer Review File are included in the article's Creative Commons license, unless indicated otherwise in a credit line to the material. If material is not included in the article's Creative Commons license and your intended use is not permitted by statutory regulation or exceeds the permitted use, you will need to obtain permission directly from the copyright holder.
To view a copy of this license, visit https://creativecommons.org/licenses/by/4.0/

From: Din Ping Tsai
To: Reviewers, Nature Communications
Subject: Response to Reviewers' Comments

Title: Varifocal Alvarez metalens array for adaptive light-field imaging
Corresponding Author: Din Ping Tsai; City University of Hong Kong

We thank all the Reviewers for their comments and constructive advice. We are pleased to have the opportunity to respond to the Reviewers' comments and revise the manuscript. Below are our responses point-by-point and revisions in the manuscript (Highlighted) for all Reviewers' comments and questions.

For Reviewer 1:

General Comment:

This manuscript describes a pioneering and elegantly designed varifocal Alvarez metalens array (AMA) that effectively bridges the gap between flat optics and computational imaging. By integrating a tunable metasurface array into a plenoptic architecture, the authors have achieved a significant milestone in dynamic optical adaptability. The demonstration of continuous focal length tuning via simple lateral displacement, coupled with the sophisticated MultiLensFusion algorithm, represents a robust and innovative approach to "all-in-focus" 3D scene reconstruction. The system-level implementation is impressive and well-executed, offering a compelling vision for next-generation light-field platforms. While the conceptual framework is strong, several technical aspects regarding characterization and physical modeling require further clarification to ensure the manuscript meets the high standards of rigor for publication.

Response:

We thank the reviewer for the highly encouraging and positive evaluation of our work. In response to the constructive feedback regarding characterization and physical modeling, we have provided detailed clarifications, additional simulation data, and expanded technical descriptions in the revised manuscript. We addressed the comments point-to-point as follows.

Specific Comments:

1. In the resolution experiments, the resolvable spatial frequency decreases significantly for distant objects, which is consistent with the system geometry and angular sampling constraints. The manuscript would benefit from a more explicit quantitative analysis of the trade-off between spatial resolution and angular sampling in the plenoptic configuration. Providing theoretical limits or analytical estimates would improve the completeness and rigor of this evaluation.

Response:

We thank the reviewer for this constructive suggestion. We agree that the spatial-angular trade-off in the plenoptic configuration should be stated more explicitly and quantitatively. In the revised manuscript, we have clarified this point using the theoretical framework already introduced in the Supplementary Information.

Specifically, the angular sampling capability of our system is characterized by the angular sampling density $|v| = |a/B|$, where $|a|$ is the distance between the main-lens image and the Alvarez metalens array, and B is the distance between the Alvarez metalens array and the image sensor (Using the symbol definitions in the Supplementary Information II), which indicates how many metalenses simultaneously capture the same point on the main-lens image plane. The spatial resolution, on the other hand, is limited by the minimum resolvable sensor-side spot size p_0, and is summarized by the image-side effective resolution ratio ε_P. In the ideal case where the sub-metalens apertures are infinitesimally small, the upper bound of ε_P is $1/|v|$. Therefore, the angular sampling $|v|$ inevitably comes at the expense of effective spatial resolution ratio ε_P, which quantitatively reflects the intrinsic trade-off in the plenoptic architecture.

We further clarify the effect of object distance on this trade-off. As the object is placed farther away in our system, the angular sampling density $|v|$ decreases, meaning that fewer metalenses simultaneously observe the same scene point. As a result, the angular sampling becomes sparser, reducing disparity sensitivity and weakening the angular support available for depth estimation and reconstruction. At the same time, the experimentally measured resolvable spatial frequency also decreases for distant objects. We now clarify that this

measured quantity corresponds to the object-space spatial resolution of the overall system, which is influenced not only by the image-side effective resolution ratio but also by the system geometry and the reduced angular sampling support. Therefore, the observed drop in resolvable spatial frequency for distant objects is consistent with both the plenoptic sampling constraint and the imaging geometry of the system.

To relate this trade-off to the resolution experiment in Fig. 4, it is important to distinguish the image-side effective resolution ratio ε_P from the object-space resolvable spatial frequency F_{sp}. The first-order lateral magnification M_L can be calculated using system parameters. Then, the object-space minimum resolvable feature size can be estimated as

$$\Delta_{obj} = \frac{p_0}{\varepsilon_p M_L},$$

and the corresponding object-space resolvable spatial frequency is

$$F_{sp} = \frac{\varepsilon_p M_L}{2p_0}.$$

This expression shows that the experimentally measured object-space resolution decreases for distant objects because the reduction in magnification M_L dominates the trend, even though the image-side effective resolution ratio ε_P may improve in the Galilean-type plenoptic configuration.

We have added this clarification and the corresponding analytical estimate in the **Supplementary Information II**:

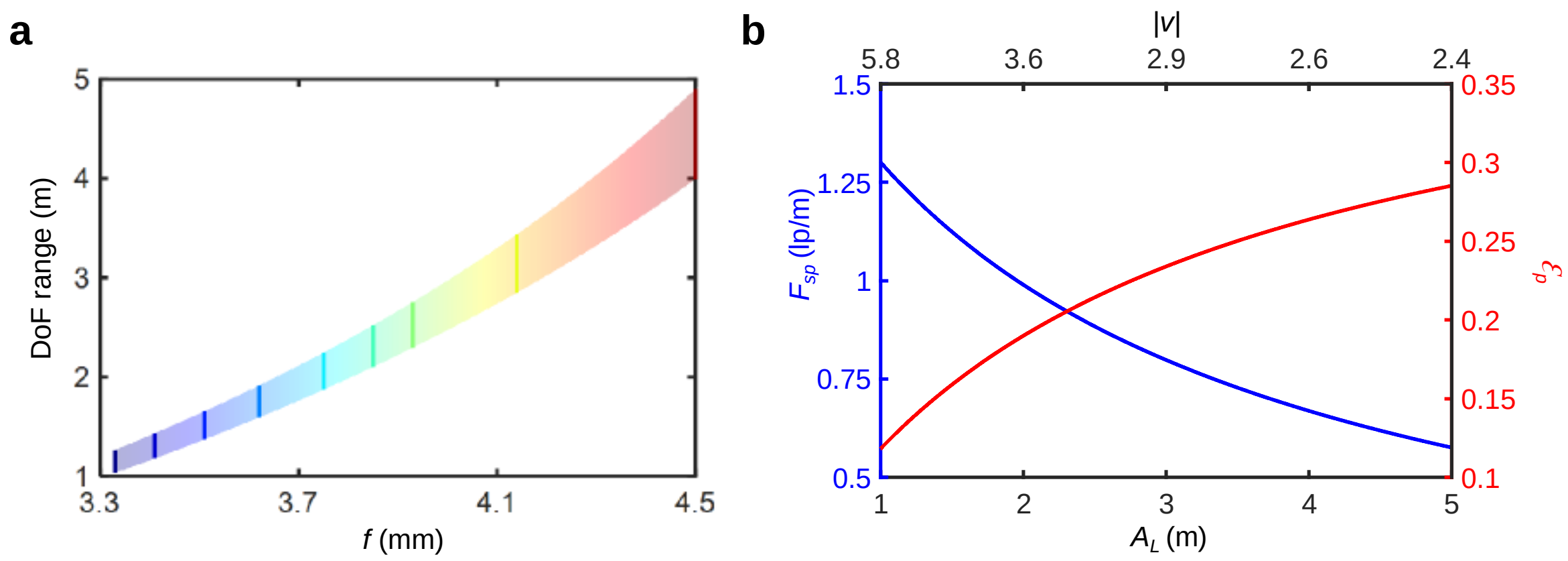

Figure S7 Optical performance of the ALI. a DOF range as a function of the sub-metalens focal length. **b** Object-space resolvable spatial frequency F_{sp} (blue) and image-side effective resolution ratio ε_P (red) vary with object distance A_L and its corresponding angular sampling

density $|v|$, respectively. **(Page 12 in Supplementary Information)**

Page 12 in the Supplementary Information:

The image-side effective resolution ratio of an ALI, denoted as ε_P, is defined as the ratio between the system′s resolution and the native resolution of the image sensor.

Pages 12-13 in the Supplementary Information:

where $|v| = |a/B|$ represents the angular sampling density, which quantifies how many metalenses simultaneously record the same point on the main-lens image plane. As shown in Fig. S7b, there is a trade-off between the spatial resolution ratio ε_P and angular sampling density $|v|$ in this plenoptic configuration. Because the focal length of the designed metalens array f can be continuously varied within a certain range, we can always achieve the minimal resolvable spot size on the image sensor. Then the effective resolution ratio can be written as

$$\varepsilon_p = \frac{1}{|v|}\frac{p}{p_0}. \qquad \text{(S1)}$$

Equation S7 shows that the angular sampling density $|v|$ inevitably comes at the expense of the effective spatial resolution ratio ε_P.

It is important to distinguish the image-side effective resolution ratio ε_P from the object-space resolvable spatial frequency F_{sp}, which is related to the resolution experiment in Fig. 4. With the magnification $M_L = (B_L\text{-}B+|a|)/A_L$, the object-space minimum resolvable feature size can be estimated as $\Delta_{obj} = p_0 / \varepsilon_p M_L$. The corresponding object-space resolvable spatial frequency is

$$F_{sp} = \frac{1}{2\Delta_{obj}} = \frac{\varepsilon_p M_L}{2p_0}. \qquad \text{(S2)}$$

Equation S8 shows that the experimentally measured object-space resolution decreases for distant objects because the reduction in magnification M_L dominates the trend, even though the image-side effective resolution ratio ε_P may improve in the Galilean-type plenoptic configuration.

2. In the Introduction, the manuscript groups Refs. 34-43 under MEMS-based tunable metalenses. However, only Ref. 34 and Ref. 39 employ genuine MEMS actuation. The remaining references rely on external mechanical translation or non-MEMS mechanisms. This classification is therefore not entirely accurate.

Response:

We thank the reviewer for pointing out this inaccuracy and for their careful reading of our manuscript. The reviewer is absolutely correct that only Refs. 34 and 39 utilize genuine MEMS actuation, whereas the other cited works rely on macroscopic mechanical translation.

To ensure precise classification, we have revised the corresponding statement in the **Introduction** to improve its rigor and clarity:

Page 2 in the Main Text:

Existing varifocal metalenses employ tunable mechanisms such as flexible substrates[22,23], phase change materials[24-28], thermoelectric materials[29-31], optical Kerr effect[32,33], and external motion systems[34-45]. Among them, external-motion-systems-based varifocal designs are particularly notable for their wide tuning range and high precision in focal length adjustment, which are achieved through continuous relative motion between two constituent metasurfaces. The overall systems can be further miniaturized by integrating metasurfaces with micro-electromechanical systems (MEMS)[34,39]. Moiré metalenses and Alvarez metalenses represent two potential architectures for MEMS-based varifocal metalenses, driven by relative rotational motion and relative lateral displacement motion, respectively.

3. The authors state that the metasurface patterns are inscribed approximately 100 μm beneath the wafer surface to provide subsurface protection. However, the high-magnification SEM images shown in Fig. 1e and Fig. S1e provide extremely clear details of the nanostructures. Given that standard SEM imaging is a surface-sensitive technique and cannot penetrate a 100 μm silica layer, the manuscript should clarify how these SEM images were obtained. Specifically, the authors should indicate whether the images were acquired from a cross-sectioned sample or from a dedicated surface-patterned test wafer.

Response:

We thank the reviewer for pointing out this lack of clarity. We agree that standard SEM imaging is a surface-sensitive technique and cannot image nanostructures buried beneath a 100 μm silica overlayer directly. To obtain these images, we lapped and polished the laser-processed glass to expose the laser-modified region, and etched the exposed surface with a 1% hydrofluoric (HF) acid solution for 5 s. The SEM images of the etched surfaces were then captured. Therefore, these images were not directly obtained by imaging through the 100-μm glass layer, nor from a separate surface-patterned test wafer, but from the processed sample after post-preparation that revealed the buried structures.

We have added the explanation in **Methods**:

Page 12 in the Main Text:

For nano-structural characterization, the laser-processed glass was lapped and polished to reveal the modified region, followed by chemical etching in a 1% hydrofluoric (HF) acid solution for 5 s. The etched surfaces were subsequently imaged using a scanning electron microscope (SEM, Zeiss EVO 50).

4. The authors state that "The metasurfaces are embedded within one-inch-diameter silicon wafers." However, throughout the rest of the manuscript and the "Methods" section, the substrate is referred to as fused silica. Since silicon is opaque at the operating wavelength (532 nm) and the laser-writing process described is specifically for transparent dielectrics, this appears to be a significant typographical error.

Response:

We thank the reviewer for their meticulous reading and for catching this significant typographical error. We apologize for this careless oversight. Furthermore, we have thoroughly proofread the entire manuscript and the Supplementary Information to ensure that the terminology for the substrate material is strictly consistent and accurate throughout.

We have corrected this error in the **Results**:

Page 4 in the Main Text: The metasurfaces are embedded within one-inch-diameter silica wafers with a thickness of 1 mm.

5. The authors specify that the total propagation distance between the two layers l_g is about 200 μm. While the Rayleigh-Sommerfeld diffraction method is used to compensate for this separation, the manuscript does not provide a formal justification for selecting this specific depth. The authors are encouraged to clarify the rationale behind the choice of 200 μm, including whether it is determined by fabrication constraints, mechanical considerations, or optical performance optimization. In principle, the Alvarez mechanism relies on the near-field superposition of two phase profiles. As the l_g increases, diffraction may introduce phase blurring and spatial crosstalk between the 345 μm pitch sub-lens regions. A quantitative discussion or simulation addressing the functional limit of l_g and its impact on PSF quality or focal length accuracy would significantly strengthen the physical justification of the design.

Response:

We appreciate the reviewer's insightful comment regarding the propagation distance l_g = 200 μm between the two metasurface layers. We agree that a large l_g can introduce diffraction-induced phase blurring and spatial crosstalk. In principle, a smaller l_g is preferable for suppressing these effects. However, the minimum achievable spacing in our design was constrained by the current fabrication and assembly process, and 200 μm was therefore chosen as the smallest practically realizable l_g.

The gap l_g introduced phase distortion. As light propagates through the first metasurface, it is modulated by a cubic phase profile function (Eq. 1). In the metasurface regions characterized by a large phase gradient, the optical rays experience a more pronounced angular deflection. Consequently, after propagating across the gap l_g, these rays significantly deviate from their ideal illumination areas on the second metasurface, which ultimately leads to a departure from their ideal trajectories. As l_g increases, the phase distribution of the light reaching the second metasurface exhibits a greater deviation from the ideal phase profile, as shown in Fig. S4a. To mitigate the impact of this unavoidable gap l_g, one effective strategy is to reduce the numerical aperture of the metalens design. A smaller numerical aperture decreases the required phase gradient on the first metasurface, which consequently suppresses the ray deflection angle and alleviates the deviation caused by the propagation gap.

Fabricating the nanostructures within the bulk substrate dictated the minimum l_g.

During the fabrication, positioning the nanostructures too close to the substrate surface can result in surface ablation, microcracks, or even localized material detachment at the air-glass interface, due to the high peak intensity of the laser and the rapid plasma expansion it induces. To achieve high-quality optical surfaces and minimize unintended surface damage, a distance of 100 μm between the fabricated nano-structures and the wafer surface was maintained to confine the laser-induced modifications strictly within the bulk of the silica glass. Consequently, the uniformity of the nanostructure morphology and the stability of its optical functionality are preserved. This approach not only enhances the reproducibility of the fabrication process but also provides a reliable foundation for the subsequent integration of high-precision optical components. Therefore, the minimum distance between the two metasurfaces l_g is 200 μm. This distance could be further reduced through post-process grinding and re-polishing, as a shorter l_g is generally preferred as it minimizes phase distortion caused by light propagation, thereby reducing the aberration and deformation of the PSF.

To further strengthen the physical justification of this design, we simulated the intensity distribution of the focus spots with different l_g values before lateral displacement (displacement ratio = 0), as shown in Fig. S4a. At smaller l_g, the intensity distribution of the focal spot on the focal plane closely approximates an ideal Airy disk. However, as the gap increases, the sidelobes in the focal plane become increasingly pronounced and undergo severe distortion. Concurrently, the focal spot becomes elongated along the *z*-axis, adopting a profile that resembles that of an Airy beam. For comparative analysis, we also simulated the focal spot intensity distribution after the lateral displacement (displacement ratio = 1), as shown in Fig. S4b. The metalens array features a longer focal length and a correspondingly smaller numerical aperture (0.038). We calculated the focal lengths and the focusing efficiencies with different l_g values to evaluate the impact of l_g on focal length accuracy and PSF quality, as shown in Fig. S4c and S4d, respectively. The focusing efficiency decreases substantially (24% at displacement ratio = 0, 11% at displacement ratio = 1) with the l_g increases, while the focal length changes slightly (<5%).

We have added explanations in the **Results** and **Supplementary Information I**:

Page 15 in the Main Text:

In this work, l_g is approximately 200 μm, which is constrained by the current fabrication and assembly process, as detailed in Supplementary Information I.

Pages 6-8 in the Supplementary Information:

In principle, the Alvarez mechanism relies on the near-field superposition of two phase profiles. The gap l_g inevitably introduces diffraction-induced phase blurring and spatial crosstalk. Specifically, regions on the first metasurface with large phase gradients impart pronounced angular deflections to the incident rays. As these rays propagate across l_g, they deviate from their ideal trajectories before reaching the second metasurface.

To quantitatively assess this functional limit, the PSF and focusing efficiency across varying l_g values are simulated, as shown in Fig. S4. At minimal gap distances, the focal spot closely resembles an ideal Airy disk. However, as l_g increases, the sidelobes become increasingly prominent, and the focal spot elongates along the optical axis, as shown in Figs. S4a and S4b. While the focal length changes slightly (<5%), the focusing efficiency notably decreases (>10%), as shown in Figs. S4c and S4d, respectively. In addition, our analysis demonstrates that reducing the numerical aperture effectively mitigates this degradation. A smaller numerical aperture requires a reduced phase gradient, which inherently suppresses the ray deflection angles and improves the system's tolerance to the gap l_g.

The gap distance l_g is fixed at 200 μm in our current configuration, which is primarily dictated by fabrication constraints. During the femtosecond laser direct writing process, the high peak intensity and subsequent rapid plasma expansion can cause surface ablation, microcracks, or localized material detachment if the structures are positioned too close to the air-glass interface. To ensure the uniformity of the nanostructures and preserve the optical quality of the surface, a safety margin of 100 μm below the wafer surface was strictly maintained. Consequently, assembling two such substrates inherently yields a minimum gap of $l_g = 200$ μm. While this gap guarantees structural stability, it could be further reduced in future iterations through post-process grinding and polishing to minimize propagation-induced phase distortion.

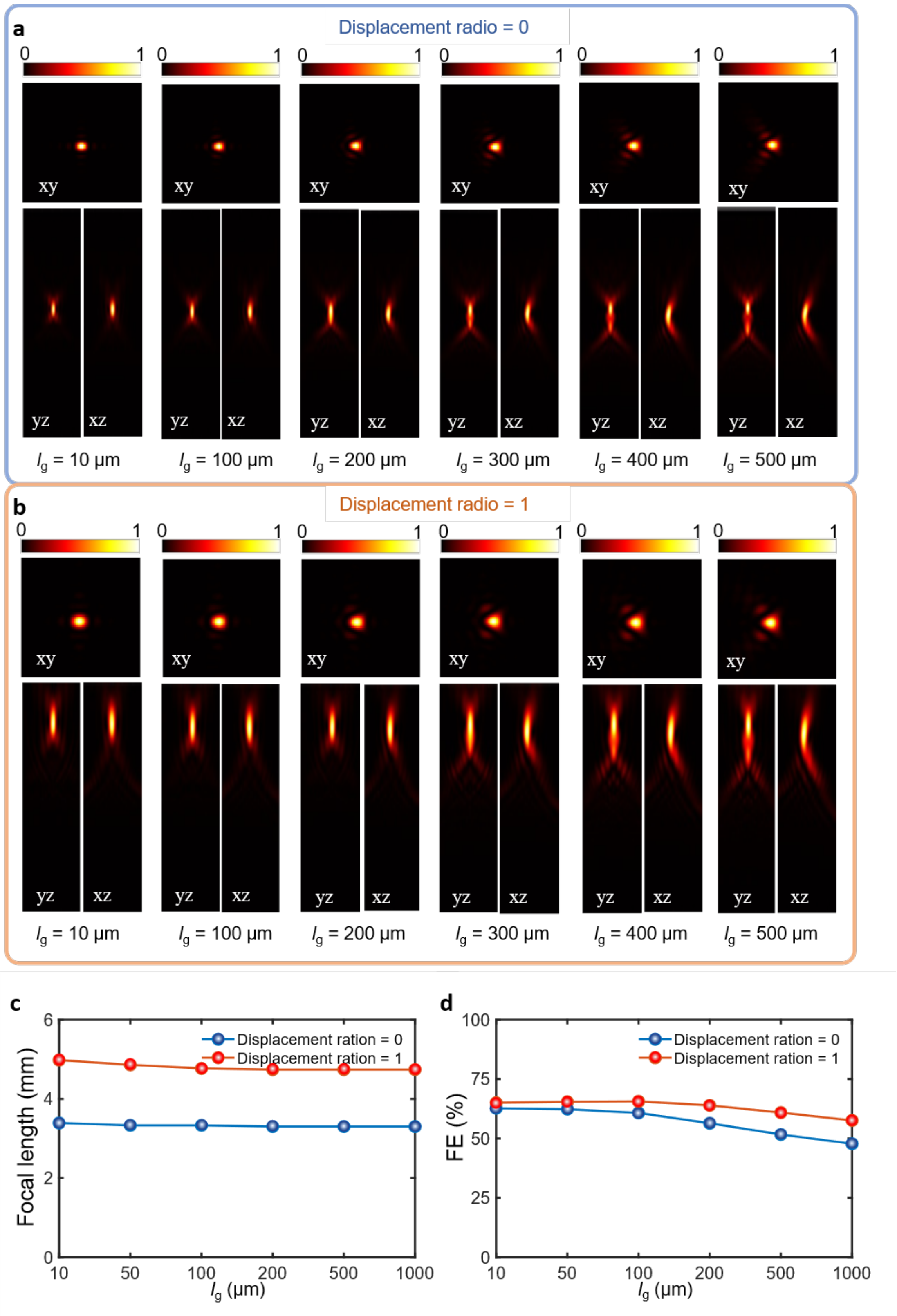

Fig. S4 Simulated focusing performance of the Alvarez metalens array with different gap length l_g. Simulated intensity distribution of the focus spots in xy-, xz-, and yz-plane when the displacement ratio is **a** 0 and **b** 1, respectively. **c** Focal length and **d** focusing efficiency (FE) vary versus l_g with different displacement ratios. **(Page 7 in the Supplementary Information)**

6. The proposed AMA does not incorporate an achromatic design. Theoretically, a metasurface with a fixed phase profile should exhibit significant chromatic aberration across the visible spectrum. However, the imaging results presented in the manuscript (especially the full-color scenes in Fig. 4 and Fig. 5) do not show severe color fringing or dispersion-induced blurring. Please provide an explanation for this observation.

Response:

We thank the reviewer for this important comment. We agree that the proposed Alvarez metalens array (AMA) is not achromatic. In fact, slight color fringing is present in the raw images. For this reason, the resolution target image in Fig. 4 was converted to grayscale, so that the evaluation of spatial resolution would not be visually affected by residual chromatic artifacts.

The absence of severe visible color fringing in Fig. 5 does not indicate achromatic behavior of the AMA; rather, in the present optical configuration, the AMA mainly introduces longitudinal chromatic defocus, whereas lateral chromatic misregistration is suppressed because each sub-metalens operates over a small field angle and at a low numerical aperture (NA). In our system, the achromatic main lens first forms a color-corrected intermediate image of the scene, and each sub-metalens operates in a highly paraxial regime. Since each sub-metalens captures only a small local portion of the intermediate image, the effective field angle is small. As a result, the chromatic error introduced by the AMA is dominated by longitudinal chromatic aberration, rather than strong transverse chromatic aberration. This greatly suppresses visible lateral color fringing. In addition, the numerical aperture of the sub-metalenses is only 0.038-0.052. Such a small NA gives a large depth of focus (λ/NA^2), and also reduces the sensor-plane blur caused by chromatic focal shift. Therefore, although different wavelengths focus at different axial positions, the resulting sensor-plane blur remains limited in our system because the low sub-metalens NA provides a comparatively large depth of focus; accordingly, the residual chromatic aberration is manifested mainly as mild softening and weak color fringing rather than severe image degradation.

As a result, the residual chromatic aberration from the AMA is manifested mainly as slight softening and weak color fringing, rather than severe dispersion-induced image degradation.

This explains why the full-color scenes in Fig. 5 still preserve good visual quality even though the AMA itself is not achromatic. We have revised the manuscript to clarify this point more explicitly.

We have revised **the caption for Fig.4** and supplemented the explanation in the **Supplementary Information III**:

Page 7 in the Main Text:

The AMA is placed slightly away from the focal length of the achromatic main lens and the image sensor, re-imaging the main lens image and creating a sub-image array onto the image sensor.

Page 8 in the Main Text:

Grayscale sub-images of a USAF 1951 resolution target captured by the ALI system with focal lengths **a** 3.33 mm, **b** 3.75 mm, and **c** 4.50 mm, and the corresponding rendered image at depths of 1.25 m, 2.5 m, and 5 m.

The imaging results here are converted to grayscale images.

Page 23 in the Supplementary Information:

In addition, we find that the chromatic aberration in our imaging system does not severely degrade the overall image quality even before any rendering or stitching process. In our system, the achromatic main lens first forms a color-corrected intermediate image of the scene. The AMA then acts on this intermediate image as a local sampling and relay element over a short propagation distance, and each sub-metalens operates in a highly paraxial regime. Since each sub-metalens captures only a small local portion of the intermediate image, the effective field angle is small. As a result, the chromatic error introduced by the AMA is dominated by longitudinal chromatic aberration, rather than strong transverse chromatic aberration. This greatly suppresses visible lateral color fringing. Furthermore, the numerical aperture (NA) of the sub-metalenses is only 0.038-0.052. Such a small NA gives a large depth of focus ($\sim\lambda/NA^2$), and also reduces the sensor-plane blur caused by chromatic focal shift. Therefore, although different wavelengths focus at different axial positions, the corresponding wavelength-dependent blur on the sensor remains modest.

7. In Fig. 1a, the schematic of the 2×2 AMA with tunable focus spots requires improvement. The phase pattern illustrated on the metasurface does not clearly resemble an Alvarez-type cubic phase profile, which may cause confusion regarding the underlying operating principle. In addition, the red curves shown in the schematic are not explained in the figure caption or main text, and their physical meaning (e.g., focal shift, beam propagation, or displacement direction) remains unclear.

Response:

We thank the reviewer for pointing out these ambiguities. We agree that a rigorous schematic is crucial for accurately conveying the underlying operating principle of the Alvarez metalens array. We have thoroughly revised **Fig. 1a** and updated the corresponding description in the **figure caption**:

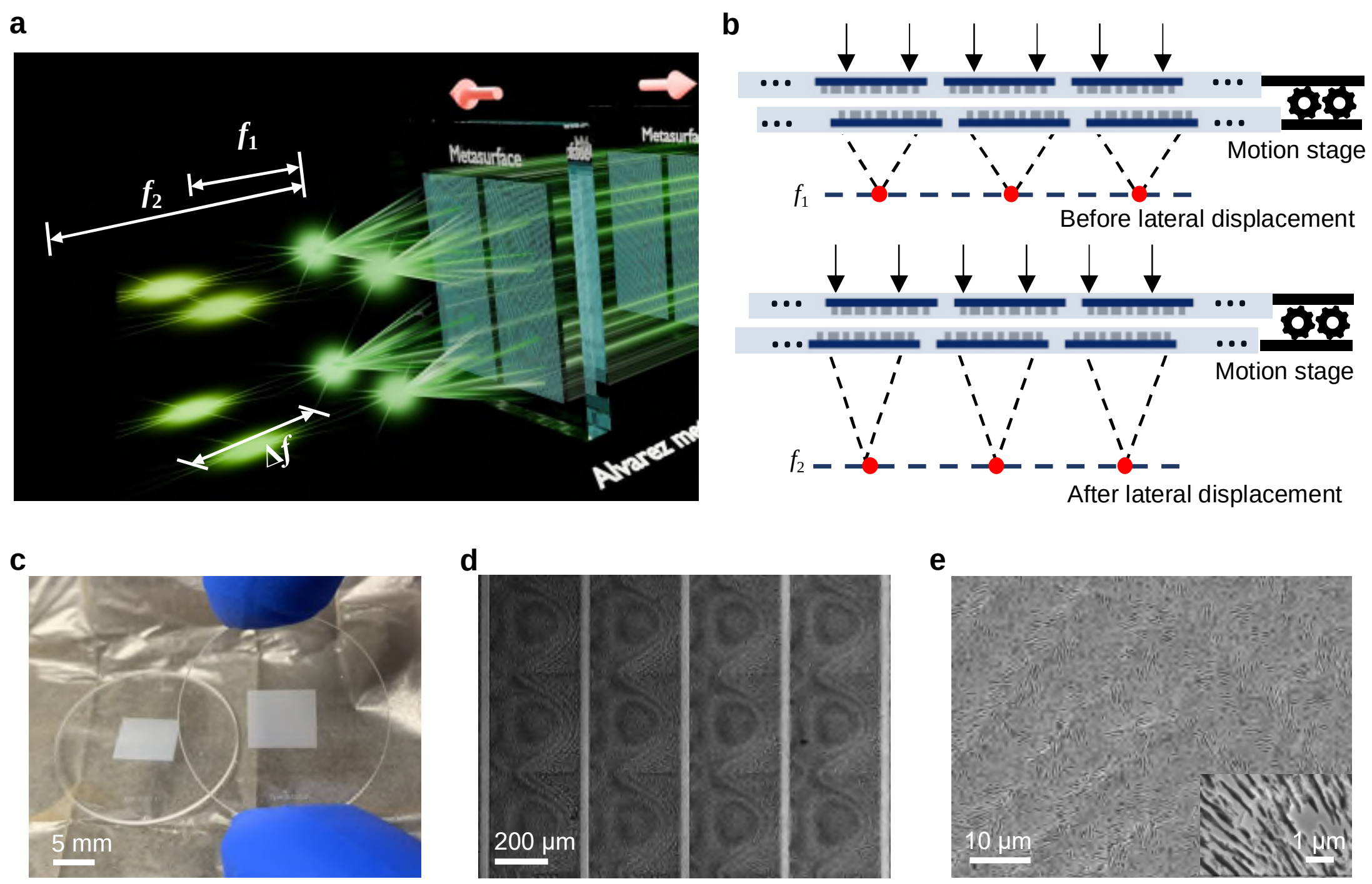

Fig. 1 Schematic diagram and sample information of the AMA. **a** Schematic of a 2×2 AMA with tunable focal lengths from f_1 to f_2. The focal shift Δf is induced by the relative lateral displacement between the metasurfaces (indicated by the red arrows). **… (Page 4 in the Main Text)**

8. The manuscript states that the metasurface structures are fabricated using femtosecond laser direct writing inside the substrate; however, the fabrication parameters are not described in sufficient detail. For clarity and reproducibility, the authors should provide the complete laser processing parameters, including laser wavelength, pulse duration, repetition rate, objective numerical aperture (NA), effective voxel size (lateral and axial), and the minimum achievable feature size. In addition, it is important to clarify how the minimum writable feature size and structural pitch compare with the operating wavelength. To achieve effective phase modulation without generating unwanted higher-order diffraction, the nanostructure period must satisfy the subwavelength condition (i.e., smaller than the working wavelength). The manuscript should therefore specify the structural pitch and provide a quantitative comparison between feature size, pitch, and operating wavelength, together with a discussion of phase sampling requirements and discretization error.

Response:

We thank the reviewer for this constructive comment. We agree that detailed fabrication parameters and a quantitative discussion of the structural dimensions are essential for reproducibility and for validating the phase modulation mechanism. We provide the complete laser processing parameters, including laser wavelength (1030 nm), pulse duration (500 fs), repetition rate (200 kHz), objective numerical aperture (0.16), effective voxel lateral size (~1.5 μm), axial size (~70 μm), and the minimum achievable feature size (~20 nm). In addition, the subwavelength features lie in the fact that the fabrication mechanism does not rely on the optical focusing resolution of the writing laser, but is instead governed by the strong nonlinear ionization and plasma dynamics induced by femtosecond laser irradiation in fused silica. The fabricated structures in this work consist of self-organized nano-gratings composed of periodically arranged nano-slots, whose geometrical scale can be precisely controlled by tuning the femtosecond laser writing parameters. The grating period is strongly dependent on the laser conditions: it decreases with increasing pulse number or density, while it increases with higher single-pulse energy. Under typical conditions, the period of such nano-gratings is approximately 350 nm, which is close to $\lambda/2n$, where λ is the wavelength of the writing laser beam (1030 nm), and n is the refractive index of silica glass at 1030 nm. Notably, each grating

period contains multiple nano-slot substructures (typically around ten), with an individual nano-slot width of approximately 20 nm, enabling subwavelength local feature dimensions. Therefore, the feature size and pitch size satisfy the subwavelength condition, and unwanted high-order diffraction is not generated.

To clarify the laser writing parameters, we added two paragraphs in the **Methods** section as follows:

Page 4 in the Main Text:

Fabrication is performed using a femtosecond-pulse laser to inscribe the metasurface patterns approximately 100 μm beneath the wafer surface[52], providing subsurface protection during both assembly and lateral displacement.

Page 11 in the Main Text:

To demonstrate the proposed concept, we designed two dielectric metasurfaces that were then fabricated by ultrafast laser writing. The laser writing experiments were carried out with a Yb: KGW femtosecond laser system (Carbide, Light Conversion Ltd.), operating at 1030 nm and delivering pulses with a 200 kHz repetition rate and a duration of 500 fs. The laser beam was focused via a 0.16 numerical aperture (NA) aspheric lens beneath the surface of a synthetic silica glass plate, which was mounted on a XYZ linear air-bearing translation stage (Aerotech Ltd.) for precise positioning. The single pulse energy was 1.5 μJ, and the focused beam radius was 2.7 μm, leading to a fluence of 6.5 J/cm^2 and an intensity of 13 TW/cm^2. During laser writing, the sample was translated at a speed of 3 mm/s, resulting in a pulse density of about 66 pulses/μm. The distance between the lines was fixed at 1 μm. A combination of a polarizer, an electro-optic modulator, and a quarter-wave plate was used to control the polarization azimuth of the laser beam. The retardance and slow-axis orientation of the birefringent modifications were quantified using a birefringence measurement system (Oosight imaging system, Hamilton Thorne) operating at 546 nm and built into an Olympus BX53 optical microscope.

Page 12 in the Main Text:

Moreover, during high-intensity femtosecond laser writing, tightly focused ultrashort pulses generate a high-density free-electron plasma inside silica glass. The interaction between the incident light field and the plasma produces a periodic electric-field modulation, which gradually stabilizes under multi-pulse irradiation and leads to the formation of self-assembled nano-gratings[56]. Because the grating period is far below the incident wavelength, direct writing of subwavelength structures can be achieved. The period of such nano-gratings was approximately 350 nm, which was close to $\lambda_w/2n$, where λ_w was the wavelength of the writing laser beam (1030 nm), and n was the refractive index of silica glass at 1030 nm.

Page 15 in the Main Text:

52. Sakakura, M., Lei, Y., Wang, L., et al. Ultralow-loss geometric phase and polarization shaping by ultrafast laser writing in silica glass. *Light Sci. Appl.*, **9**, 15 (2020).

56. Shimotsuma, Y., Kazansky, P. G., Qiu, J., Hirao, K. Self-organized nanogratings in glass irradiated by ultrashort light pulses. *Phys. Rev. Lett.*, **91**, 247405 (2003).

Page 3 in the Supplementary Information:

The fundamental reason that these structures can achieve sub-100 nm feature sizes lies in the fact that the fabrication mechanism does not rely on the optical focusing resolution of the writing laser, but is instead governed by the strong nonlinear ionization and plasma dynamics induced by femtosecond laser irradiation in fused silica. The fabricated structures in this work consist of self-organized nano-gratings composed of periodically arranged nano-slots, whose geometrical scale can be precisely controlled by tuning the femtosecond laser writing parameters. The grating period is strongly dependent on the laser conditions: it decreases with increasing pulse number, while it increases with higher single-pulse energy. Under typical conditions, the period of such nano-gratings is approximately 350 nm. Notably, each grating period contains multiple nano-slot substructures (typically around ten), with an individual nano-slot width of approximately 20 nm, enabling sub-100 nm local feature dimensions.

9. In Figure S3, the caption and accompanying description appear incomplete. Specifically, Fig. S3b is not described or discussed in the text. The Supplementary Information should be revised to ensure that all subfigures, including Fig. S3b, are properly described, clearly labeled, and consistently referenced. This would improve clarity and avoid potential ambiguity.

Response:

We thank the reviewer for his/her careful reading and for pointing out this oversight. We apologize for the missing description of Figure S3 in the initial Supplementary Information. We have double-checked the main manuscript and the Supplementary Information to ensure that all subfigures are accurately and consistently referenced throughout the text.

To address this and avoid any potential ambiguity, we have carefully revised the **caption for Figure S3** and the corresponding description:

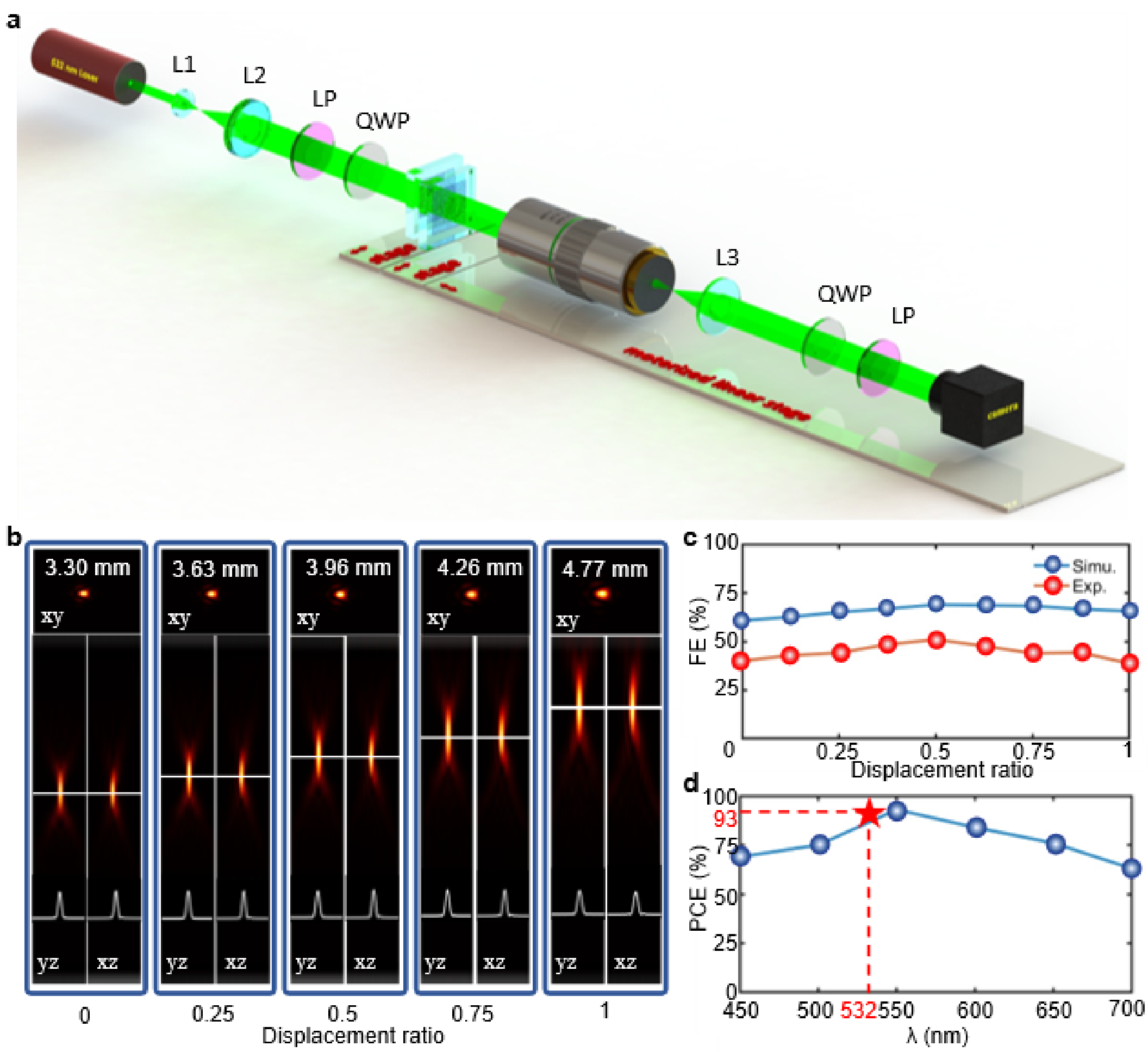

Figure S3 Point spread function (PSF) measurement setup and focusing performance of

the Alvarez metalens array (AMA). a Schematic of the PSF measurement setup. **b** Intensity distribution of the focus spots in *xy*-, *xz*-, and *yz*-plane with different displacement ratios. Corresponding intensity profiles at the focal planes are shown below each distribution. **c** Focusing efficiency (FE) as a function of displacement ratio. **d** Measured polarization conversion efficiency (PCE) of the nanostructures. **(Page 6 in the Supplementary Information)**

Page 5 in the Supplementary Information:

The three-dimensional intensity distribution of the focal region is then captured, as shown in Fig. S3b. We set f_1 = 3.4 mm, f_2 = 5 mm, d_{max} = 69 μm, l_g = 200 μm, λ = 532 nm. According to Eqs. 1 and 2, the simulated focal length varies from 3.30 mm to 4.77 mm with the lateral displacement, in good agreement with the experimental results presented in Fig. 2f.

For Reviewer 2:

General Comment:

This manuscript presents a compelling demonstration of a varifocal Alvarez metalens array (AMA) integrated into a plenoptic imaging system. The concept of achieving dynamic optical adaptability through lateral displacement is innovative, and the combination with a MultiLensFusion algorithm to achieve an extended depth-of-field is significant. The work is well-structured and falls within the scope of the journal. However, several critical points need to be addressed before publication.

Response:

We thank the reviewer for the positive comments and constructive suggestions on our manuscript. We have carefully addressed each of the critical points as follows. We hope these improvements adequately address your concerns.

<u>Specific Comments:</u>

1. The introduction provides a good overview but omits key references highly relevant to the work. The authors should discuss and cite recent advances in "Polarization-separating Alvarez metalenses" and "Large Area Tunable Alvarez Metalenses via Stepper Photolithography." Including these will better position the novelty of this work.

Response:

We thank the reviewer for bringing these excellent and highly relevant recent advances to our attention. We agree that including these works provides a more comprehensive overview of the rapid progress in Alvarez metalenses. Incorporating the methodologies from these studies could further expand the functionalities of our designed device.

We have added these key references and discussed them in the revised **Introduction, Discussions, and References**:

Page 2 in the Main Text:

Existing varifocal metalenses employ tunable mechanisms such as flexible substrates[22,23], phase change materials[24-28], thermoelectric materials[29-31], optical Kerr effect[32,33], and external motion systems[34-45].

Page 11 in the Main Text:

The AMA can also incorporate polarization-dependent dual-focus capabilities while maintaining continuous varifocal operation[44].

Such miniaturized devices hold strong potential for 3D reconstruction, parallel nanofabrication[45,54], and adaptive optics.

Page 14 in the Main Text:

44. Hada, M., Adegawa, H., Aoki, K., et al. Polarization-separating alvarez metalens. Opt. Express, 32, 6672-6683 (2024).

45. Colburn, S., Zhan, A., Majumdar, A. Large area tunable alvarez metalens via stepper photolithography. Conference on Lasers and Electro-Optics. San Francisco, CA, USA, IEEE, 1-2 (2018).

2. The author mentioned in lines 116 and 305 that the metasurface was fabricated from silicon and fused silica, respectively, which is contradictory. It is suggested to modify the expression. Besides, the metasurface pattern is embedded approximately 100 micrometers below the sample surface. How was the SEM image captured?

Response:

We thank the reviewer for their meticulous reading and for catching this significant typographical error. We apologize for this careless oversight. Furthermore, we have thoroughly proofread the entire manuscript and the Supplementary Information to ensure that the terminology for the substrate material is strictly consistent and accurate throughout. In addition, we understand the reviewer's concern regarding the SEM imaging of structures embedded within the transparent and insulating silica substrate. We lapped and polished the laser-processed glass, and etched the exposed surface with a 1% hydrofluoric (HF) acid solution for 5 s. Then, the SEM images of the etched surfaces were captured.

We have corrected this error in the revised manuscript and added the explanation in **Methods:**

Page 4 in the Main Text:

The metasurfaces are embedded within one-inch-diameter silica wafers with a thickness of 1 mm.

Page 12 in the Main Text:

For nano-structural characterization, the laser-processed glass was lapped and polished to reveal the modified region, followed by chemical etching in a 1% hydrofluoric (HF) acid solution for 5 s. The etched surfaces were subsequently imaged using a scanning electron microscope (SEM, Zeiss EVO 50).

3. The relationship between the physical dimensions of the nanostructures shown in the SEM image and the achieved phase profile is not explained. What is the morphology of the micro-nano structure in the depth direction?

Response:

We thank the reviewer for pointing out this insufficient explanation. We agree that clarifying the relationship between the physical dimensions, the depth morphology, and the achieved phase profile is essential for understanding the light-matter interaction in our metasurface.

The achieved phase profile is modulated by the Pancharatnam-Berry (PB) phase, which is decided by the rotation angle θ of the nano-gratings. **As described in the Supplementary Information (Page 3)**, the induced PB phase is -2θ. The local phase profile is encoded by the orientation angle of the anisotropic nano-gratings through the PB phase, whereas the structural dimensions, including the groove width, pitch, and effective thickness, determine the birefringent retardation and are optimized to approach the half-wave condition for high polarization-conversion efficiency. By dynamically rotating the polarization of the writing femtosecond laser, we control the local orientation of the nano-gratings to impart the desired cubic phase profile of the Alvarez mechanism for circularly polarized incident light.

Regarding the morphology in the depth direction, the nanostructures form an elongated ellipsoid. This morphology is dictated by the 3D focal volume of the objective lens used for the femtosecond laser direct writing. Because the axial Rayleigh length of the focused Gaussian beam is larger than its lateral waist, the induced plasma and subsequent nanograting self-assembly extend along the beam propagation axis. Figure S1f shows the morphology of the nanostructures in the depth direction, which are fabricated by a single focused laser beam.

We have added a comprehensive explanation of this mechanism in the **Supplementary Information I** and have revised **Figure S1**:

Page 3 in the Supplementary Information:

In terms of functionality, the nanostructures operate based on an effective birefringent mechanism, with the design target of achieving a π phase retardation, corresponding to a half-wave plate response. Under this condition, precise birefringent retardation is achieved by

controlling the anisotropic structural parameters of the nano-gratings, including nano-slot density, morphological uniformity, and effective birefringence, as well as the effective propagation thickness. When the phase retardation reaches exactly π, complete conversion of the incident linear polarization state can be realized, corresponding to an ideal polarization conversion efficiency approaching 100%. Therefore, the optimization of fabrication accuracy and operational efficiency is intrinsically unified in the precise control of the self-organized nanostructure uniformity and the resulting effective birefringent phase retardation, ensuring that the device performance closely approaches the ideal half-wave plate condition for efficient optical field manipulation.

These anisotropic nano-gratings manipulate the Pancharatnam-Berry (PB) phase of the incident light. …

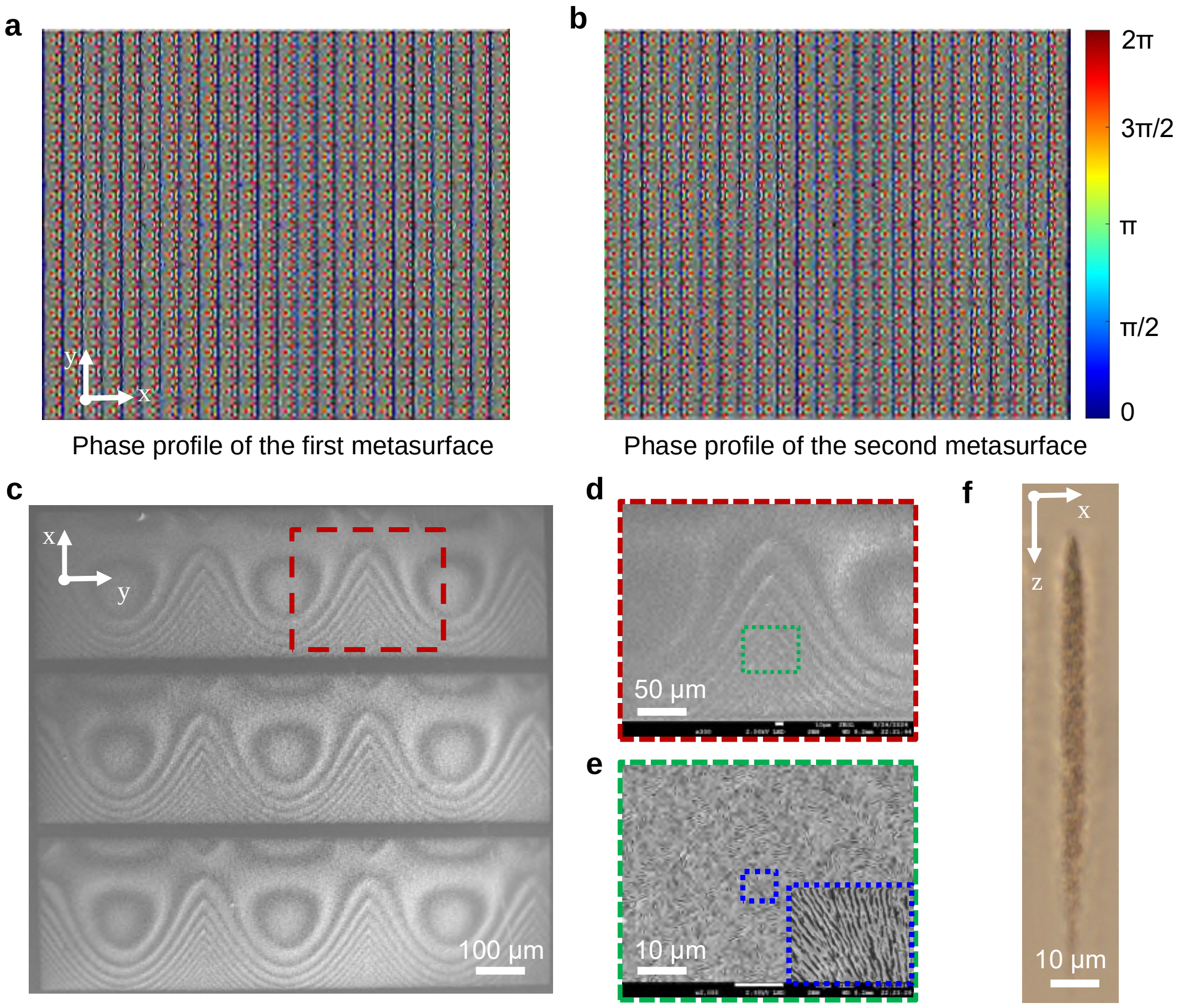

Figure S1 Phase profiles and images of the fabricated metasurfaces. The phase profiles of **a** the first metasurface and **b** the second metasurface. Scanning electron microscope (SEM) images of the fabricated metasurface with **c** 50 X, **d** 330 X, and **e** 2000 X magnification,

respectively. **f** Morphology of the nanostructures in the depth direction observed by an optical microscope. **(Page 2 in the Supplementary Information)**

Page 3 in the Supplementary Information:

Regarding the morphology in the depth direction, the nanostructures form an elongated ellipsoid. This morphology is dictated by the 3D focal volume of the objective lens used for the femtosecond laser direct writing. Because the axial Rayleigh length of the focused Gaussian beam is larger than its lateral waist, the induced plasma and subsequent nanograting self-assembly extend along the beam propagation axis. Fig. S1f shows the morphology of the nanostructures in the depth direction, which are fabricated by a single focused laser beam.

4. The manuscript mentions laser writing, but the structures in Fig. 1e appear to have sub-100 nm features, which is challenging for standard laser lithography. How these high-resolution structures were fabricated and imaged. How to control the fabrication accuracy of the structure and optimize its working efficiency?

Response:

We thank the reviewer for this insightful comment. The fundamental reason that these structures can achieve sub-100 nm feature sizes lies in the fact that the fabrication mechanism does not rely on the optical focusing resolution of the writing laser, but is instead governed by the strong nonlinear ionization and plasma dynamics induced by femtosecond laser irradiation in fused silica. The fabricated structures in this work consist of self-organized nano-gratings composed of periodically arranged nano-slots, whose geometrical scale can be precisely controlled by tuning the femtosecond laser writing parameters. The grating period is strongly dependent on the laser conditions: it decreases with increasing pulse number or density, while it increases with higher single-pulse energy. Under typical conditions, the period of such nano-gratings is approximately 350 nm, which is close to λ/2n, where λ is the wavelength of the writing laser beam (1030 nm), and n is the refractive index of silica glass at 1030 nm. Notably, each grating period contains multiple nano-slot substructures (typically around ten), with an individual nano-slot width of approximately 20 nm, enabling sub-100 nm local feature dimensions. How to image these nanostructures has been answered in **Comment 2**.

In terms of functionality, the nanostructures operate based on an effective birefringent mechanism, with the design target of achieving a π phase retardation, corresponding to a half-wave plate response. Under this condition, precise birefringent retardation is achieved by controlling the anisotropic structural parameters of the nano-gratings, including nano-slot density, morphological uniformity, and effective birefringence, as well as the effective propagation thickness. When the phase retardation reaches exactly π, complete conversion of the incident linear polarization state can be realized, corresponding to an ideal polarization conversion efficiency approaching 100%. Therefore, the optimization of fabrication accuracy and operational efficiency is intrinsically unified in the precise control of the self-organized nanostructure uniformity and the resulting effective birefringent phase retardation, ensuring

that the device performance closely approaches the ideal half-wave plate condition for efficient optical field manipulation.

We have added two references in the **main text** and some explanations in the **Supplementary Information I**:

Page 4 in the Main Text:

Fabrication is performed using a femtosecond-pulse laser to inscribe the metasurface patterns approximately 100 μm beneath the wafer surface[52], providing subsurface protection during both assembly and lateral displacement.

Page 12 in the Main Text:

Moreover, during high-intensity femtosecond laser writing, tightly focused ultrashort pulses generate a high-density free-electron plasma inside silica glass. The interaction between the incident light field and the plasma produces a periodic electric-field modulation, which gradually stabilizes under multi-pulse irradiation and leads to the formation of self-assembled nano-gratings[56]. Because the grating period is far below the incident wavelength, direct writing of subwavelength structures can be achieved. The period of such nano-gratings was approximately 350 nm, which was close to $\lambda_w/2n$, where λ_w was the wavelength of the writing laser beam (1030 nm), and n was the refractive index of silica glass at 1030 nm.

Page 15 in the Main Text:

52. Sakakura, M., Lei, Y., Wang, L., et al. Ultralow-loss geometric phase and polarization shaping by ultrafast laser writing in silica glass. *Light Sci. Appl.*, **9**, 15 (2020).

56. Shimotsuma, Y., Kazansky, P. G., Qiu, J., Hirao, K. Self-organized nanogratings in glass irradiated by ultrashort light pulses. *Phys. Rev. Lett.*, **91**, 247405 (2003).

Pages 2-3 in the Supplementary Information:

The fundamental reason that these structures can achieve sub-100 nm feature sizes lies in the fact that the fabrication mechanism does not rely on the optical focusing resolution of the writing laser, but is instead governed by the strong nonlinear ionization and plasma dynamics induced by femtosecond laser irradiation in fused silica. The fabricated structures in this work

consist of self-organized nano-gratings composed of periodically arranged nano-slots, whose geometrical scale can be precisely controlled by tuning the femtosecond laser writing parameters. The grating period is strongly dependent on the laser conditions: it decreases with increasing pulse number, while it increases with higher single-pulse energy. Under typical conditions, the period of such nano-gratings is approximately 350 nm. Notably, each grating period contains multiple nano-slot substructures (typically around ten), with an individual nano-slot width of approximately 20 nm, enabling sub-100 nm local feature dimensions.

In terms of functionality, the nanostructures operate based on an effective birefringent mechanism, with the design target of achieving a π phase retardation, corresponding to a half-wave plate response. Under this condition, birefringent retardation is achieved by controlling the anisotropic structural parameters of the nano-gratings, including nano-slot density, morphological uniformity, and effective birefringence, as well as the effective propagation thickness. When the phase retardation reaches exactly π, complete conversion of the incident linear polarization state can be realized, corresponding to an ideal polarization conversion efficiency approaching 100%. Therefore, the optimization of fabrication accuracy and operational efficiency is intrinsically unified in the precise control of the self-organized nanostructure uniformity and the resulting effective birefringent phase retardation, ensuring that the device performance closely approaches the ideal half-wave plate condition for efficient optical field manipulation.

5. The manuscript notes a "slight deviation" between the simulated total phase and the ideal phase profile. A detailed explanation of the source of this deviation is necessary to validate the design methodology and assess its limitations.

Response:

We thank the reviewer for highlighting this critical point. We completely agree that a detailed explanation of the phase deviation is essential for validating our design methodology and comprehensively assessing its limitations. The deviation between the simulated total phase and the ideal phase profile originates from free-space propagation across the nonzero gap l_g, which breaks the ideal near-field phase superposition assumed for an Alvarez pair; this effect becomes stronger in regions with larger phase gradients and therefore increases with l_g.

As light propagates through the first metasurface, it is modulated by a cubic phase profile function (Eq. 1). In the metasurface regions characterized by a large phase gradient, the optical rays experience a more pronounced angular deflection. Consequently, after propagating across the gap l_g, these rays significantly deviate from their ideal illumination areas on the second metasurface, which ultimately leads to a departure from their ideal trajectories. As l_g increases, the phase distribution of the light reaching the second metasurface exhibits a greater deviation from the ideal phase profile. To mitigate the impact of this unavoidable gap l_g, one effective strategy is to reduce the numerical aperture of the metalens design. A smaller numerical aperture decreases the required phase gradient on the first metasurface, which consequently suppresses the ray deflection angle and alleviates the deviation caused by the propagation gap.

To further strengthen the physical justification of this design, we simulated the intensity distribution of the focus spots with different l_g values before lateral displacement (displacement ratio = 0), as shown in Fig. S4a. At smaller l_g, the intensity distribution of the focal spot on the focal plane closely approximates an ideal Airy disk. However, as the gap increases, the sidelobes in the focal plane become increasingly pronounced and undergo severe distortion. Concurrently, the focal spot becomes elongated along the *z*-axis, adopting a profile that resembles that of an Airy beam. For comparative analysis, we also simulated the focal spot intensity distribution after the lateral displacement (displacement ratio = 1), as shown in Fig. S4b. The metalens array features a longer focal length and a correspondingly smaller numerical

aperture (0.038). We calculated the focal lengths and the focusing efficiencies with different l_g values to evaluate the impact of l_g on focal length accuracy and PSF quality, as shown in Fig. S4c and S4d, respectively. The focusing efficiency decreases substantially (24% at displacement ratio = 0, 11% at displacement ratio = 1) with the l_g increases, while the focal length changes slightly (<5%).

We have added a figure and some explanations in the **Supplementary Information I**:

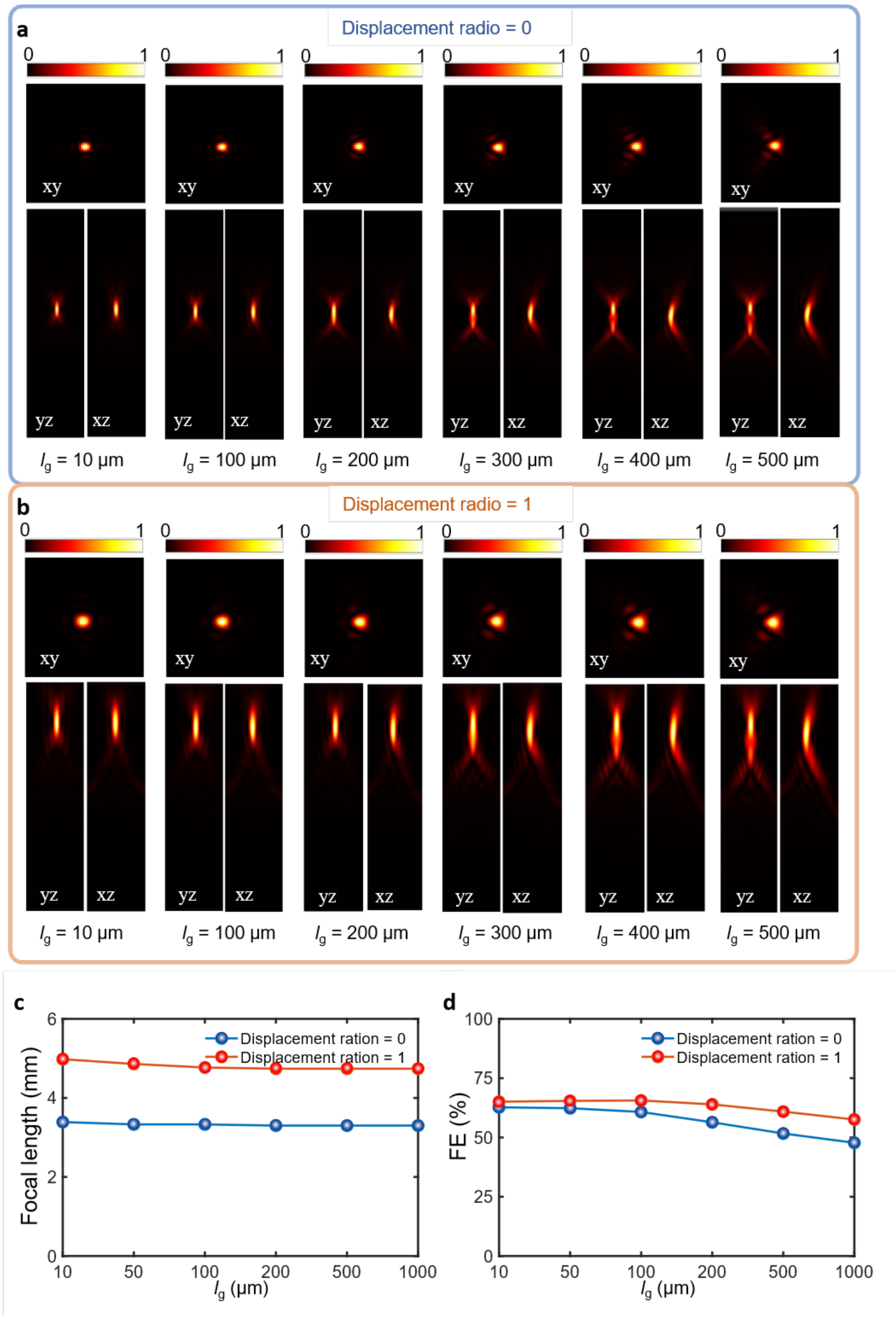

Fig. S4 Simulated focusing performance of the Alvarez metalens array with different gap

length l_g. Simulated intensity distribution of the focus spots in xy-, xz-, and yz-plane when the displacement ratio is **a** 0 and **b** 1, respectively. **c** Focal length and **d** focusing efficiency (FE) vary versus l_g with different displacement ratios. **(Page 7 in the Supplementary Information)**

Pages 6-7 in the Supplementary Information:

In principle, the Alvarez mechanism relies on the near-field superposition of two phase profiles. The gap l_g inevitably introduces diffraction-induced phase blurring and spatial crosstalk. Specifically, regions on the first metasurface with large phase gradients impart pronounced angular deflections to the incident rays. As these rays propagate across l_g, they deviate from their ideal trajectories before reaching the second metasurface.

To quantitatively assess this functional limit, the PSF and focusing efficiency across varying l_g values are simulated, as shown in Fig. S4. At minimal gap distances, the focal spot closely resembles an ideal Airy disk. However, as l_g increases, the sidelobes become increasingly prominent, and the focal spot elongates along the optical axis, as shown in Figs. S4a and S4b. While the focal length changes slightly (<5%), the focusing efficiency notably decreases (>10%), as shown in Figs. S4c and S4d, respectively. In addition, our analysis demonstrates that reducing the numerical aperture effectively mitigates this degradation. A smaller numerical aperture requires a reduced phase gradient, which inherently suppresses the ray deflection angles and improves the system's tolerance to the gap l_g.

6. The reconstructed "all-in-focus" images in Figure 5 are presented without any scale information.

Response:

We thank the reviewer for pointing out this oversight. We agree that providing physical scale information is essential for a complete and rigorous presentation of the imaging results. Because objects located at different depths in the all-in-focus image are subject to varying magnifications, placing universal scale bars directly on the reconstructed scene would clutter the visual presentation and potentially cause confusion. Therefore, to maintain visual clarity, we have supplemented the specific physical sizes for each object in **Fig. 5a** instead:

[editorial note: third party material redacted]

Fig. 5 Experiments of the ALI. a Light-field image of the fluorescent lamp boards and nesting dolls at different distances. The satellite, the planet, and the stars are at distances 1.3 m, 3.0 m, and 4.3 m, respectively. The orange, blue, pink, green, and red nesting dolls are at distances 1.3 m, 1.7 m, 2.4 m, 3.1 m, and 4.1 m. **b-d** The light-field sub-images and the corresponding rendered images with different displacement ratios 0, 0.5, and 1. **e-g** The tested disparities at different distances with different displacement ratios 0, 0.5, and 1. **h,j** The all-in-focus images of the two scenes. **i,k** Depth estimation of the scenes. **(Page 10 in the Main Text)**

7. This paper demonstrates the imaging capabilities of the Alvarez superlens array and how to further enhance the imaging resolution. What are the main limiting factors?

Response:

We thank the reviewer for pointing out this forward-looking comment. The main limiting factors of the imaging resolution in the current Alvarez-metalens-array-based plenoptic system are the spatial-angular sampling trade-off, the gap l_g, and the limited numerical aperture of each sub-metalens. These factors respectively constrain the available spatial sampling budget, introduce propagation-induced degradation of the effective phase superposition and PSF quality, and limit the diffraction-limited resolving power of each sub-image channel.

The resolution is fundamentally constrained by the spatial-angular trade-off of the plenoptic architecture. For a fixed sensor pixel budget, increasing angular sampling inevitably reduces the number of pixels available for spatial sampling, which limits the final reconstructed spatial resolution.

As shown in Fig. S4, the gap l_g between the two metasurfaces introduces propagation-induced phase blurring, which reduces the PSF quality. Reducing l_g would directly improve the imaging resolution. However, during the fabrication, positioning the nanostructures too close to the substrate surface can result in surface ablation, microcracks, or even localized material detachment at the air-glass interface, due to the high peak intensity of the laser and the rapid plasma expansion it induces. To achieve high-quality optical surfaces and minimize unintended surface damage, a certain distance between the fabricated nano-structures and the wafer surface should be maintained to confine the laser-induced modifications strictly within the bulk of the silica glass.

Furthermore, the resolution is also limited by the numerical aperture of each sub-metalens, which is currently constrained by the achievable phase gradient and fabrication tolerance of the femtosecond-laser-written nanostructures. Increasing the effective numerical aperture while maintaining accurate phase modulation would improve the diffraction-limited resolution.

Therefore, the most direct routes to further enhance the imaging resolution are to optimize the spatial-angular sampling allocation, reduce the separation gap l_g, and increase the achievable numerical aperture of the metalens array through improved design and fabrication.

We have added discussions in the **Main Text** and **Supplementary Information II**:

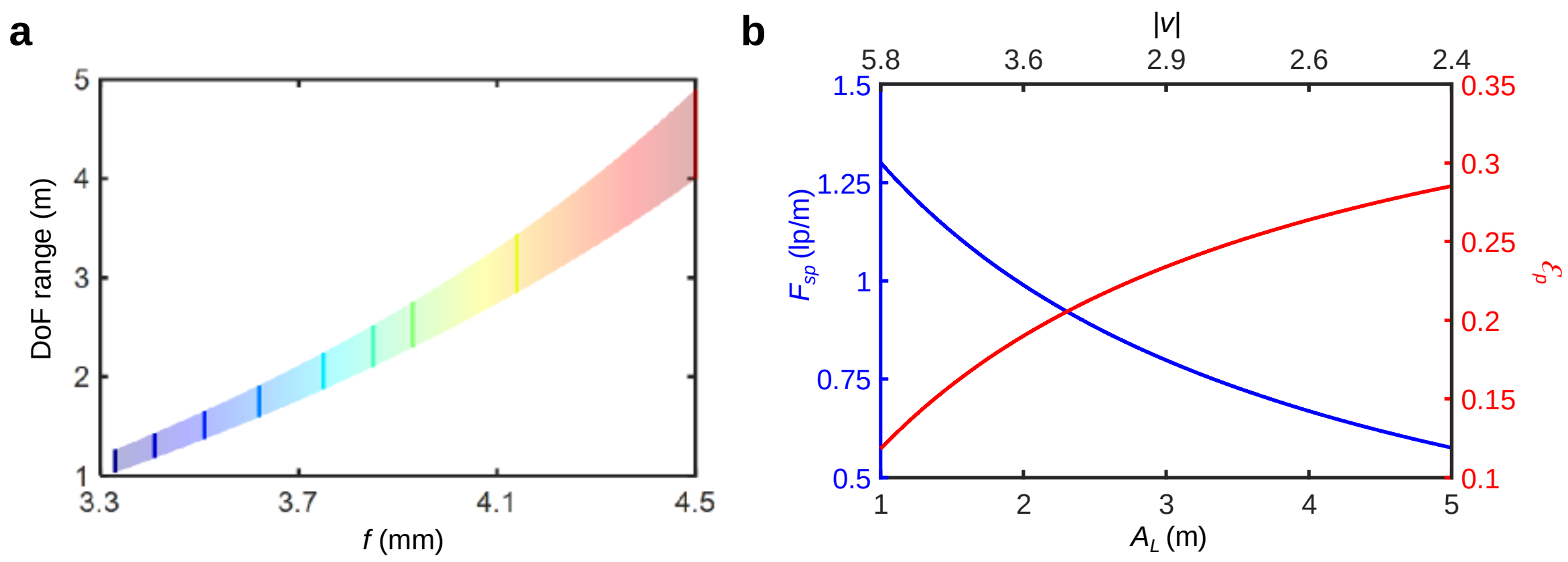

Figure S7 Optical performance of the ALI. a DOF range as a function of the sub-metalens focal length. **b** Object-space resolvable spatial frequency F_{sp} (blue) and image-side effective resolution ratio ε_P (red) vary with object distance A_L and its corresponding angular sampling density $|v|$, respectively. **(Page 12 in Supplementary Information)**

Page 12 in the Supplementary Information:

The image-side effective resolution ratio of an ALI, denoted as ε_P, is defined as the ratio between the system′s resolution and the native resolution of the image sensor.

Pages 12-13 in the Supplementary Information:

where $|v| = |a/B|$ represents the angular sampling density, which quantifies how many metalenses simultaneously record the same point on the main-lens image plane. As shown in Fig. S7b, there is a trade-off between the spatial resolution ratio ε_P and angular sampling density $|v|$ in this plenoptic configuration. Because the focal length of the designed metalens array f can be continuously varied within a certain range, we can always achieve the minimal resolvable spot size on the image sensor. Then the effective resolution ratio can be written as

$$\varepsilon_p = \frac{1}{|v|}\frac{p}{p_0}. \tag{S3}$$

Equation S7 shows that the angular sampling density $|v|$ inevitably comes at the expense of the effective spatial resolution ratio ε_P.

It is important to distinguish the image-side effective resolution ratio ε_P from the object-space resolvable spatial frequency F_{sp}, which is related to the resolution experiment in Fig. 4. With the magnification $M_L = (B_L\text{-}B+|a|)/A_L$, the object-space minimum resolvable feature size can be estimated as $\Delta_{obj} = p_0 / \varepsilon_p M_L$. The corresponding object-space resolvable spatial frequency is

$$F_{sp} = \frac{1}{2\Delta_{obj}} = \frac{\varepsilon_p M_L}{2p_0}. \tag{S4}$$

Equation S8 shows that the experimentally measured object-space resolution decreases for distant objects because the reduction in magnification M_L dominates the trend, even though the image-side effective resolution ratio ε_P may improve in the Galilean-type plenoptic configuration.

Page 8 in the Supplementary Information:

The gap distance l_{g} is fixed at 200 μm in our current configuration, which is primarily dictated by fabrication constraints. During the femtosecond laser direct writing process, the high peak intensity and subsequent rapid plasma expansion can cause surface ablation, microcracks, or localized material detachment if the structures are positioned too close to the air-glass interface. To ensure the uniformity of the nanostructures and preserve the optical quality of the surface, a safety margin of 100 μm below the wafer surface was strictly maintained. Consequently, assembling two such substrates inherently yields a minimum gap of l_{g} = 200 μm. While this gap guarantees structural stability, it could be further reduced in future iterations through post-process grinding and polishing to minimize propagation-induced phase distortion.

From: Din Ping Tsai
To: Reviewers, Nature Communications
Subject: Response to Reviewers’ Comments

Title: Varifocal Alvarez metalens array for adaptive light-field imaging
Corresponding Author: Din Ping Tsai; City University of Hong Kong

We thank all the Reviewers for their recommendation for publication.

Reviewer 1: All my concerns have been satisfactorily addressed, and I find the manuscript acceptable for publication in its current form.

Response: We thank the reviewer for the positive assessment of our revised manuscript. We are pleased that the reviewer finds our responses satisfactory and considers the manuscript acceptable for publication in its current form.

Reviewer 2: I am satisfied with the authors' responses and the modifications made to the manuscript and Supplementary Information. All my previous points have been adequately resolved. I recommend publication in its current form.

Response: We thank the reviewer for the careful evaluation of our revised manuscript and Supplementary Information. We are pleased that the reviewer is satisfied with our responses and modifications, and we appreciate the recommendation for publication.

Thanks again for helping us improve the manuscript.
